# Biodegradation, Bioassimilation and Recycling Properties of Wheat Gluten Foams

**DOI:** 10.1021/acsagscitech.4c00798

**Published:** 2025-04-04

**Authors:** Mercedes A. Bettelli, Leonardo A. Perdigón, Luyao Zhao, Pamela F. M. Pereira, Amparo Jiménez-Quero, Antonio J. Capezza, Thomas Prade, Eva Johansson, Richard T. Olsson, Mikael S. Hedenqvist, Marcos A. Sabino

**Affiliations:** † Department of Fibre and Polymer Technology, Polymeric Materials Division, School of Engineering Sciences in Chemistry, Biotechnology and Health, KTH Royal Institute of Technology, 100 44 Stockholm, Sweden; ‡ Department of Chemistry, B5IDA Research Group, Simon Bolivar University, Caracas 89000, Venezuela; § Department of Industrial Biotechnology, School of Engineering Sciences in Chemistry, Biotechnology and Health, KTH Royal Institute of Technology, 100 44 Stockholm, Sweden; ∥ Department of LIFE Sciences, Industrial Biotechnology Division, Chalmers University of Technology, 412 96 Gothenburg, Sweden; ⊥ Department of Biosystems and Technology, Swedish University of Agricultural Sciences, P.O. Box 190, 234 22 Lomma, Sweden; # Department of Plant Breeding, The Swedish University of Agricultural Sciences, P.O. Box 190, 234 22 Lomma, Sweden

**Keywords:** biobased foams, wheat gluten, biodegradation, bioassimilation, recycling

## Abstract

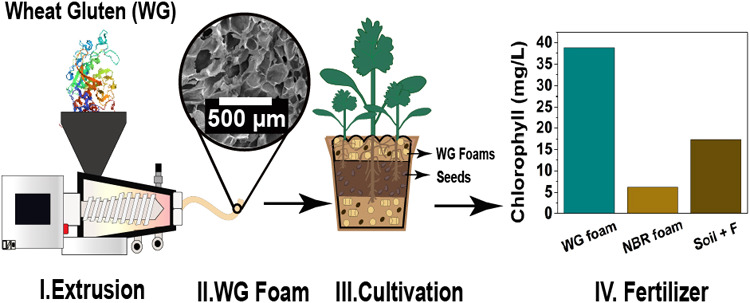

Protein-based foams are potential sustainable alternatives to petroleum-based polymer foams in e.g. single-use products. In this work, the biodegradation, bioassimilation, and recycling properties of glycerol-plasticized wheat gluten foams (using a foaming agent and gallic acid, citric acid, or genipin) were determined. The degradation was investigated at different pH levels in soil and high humidity. The fastest degradation occurred in an aqueous alkaline condition with complete degradation within 5 weeks. The foams exhibited excellent bioassimilation, comparable to or better than industrial fertilizers, particularly in promoting coriander plant growth. The additives provided specific effects: gallic acid offered antifungal properties, citric acid provided the fastest degradation at high pH, and genipin contributed with cross-linking. All three additives also contributed to antioxidant properties. Dense β-sheet protein structures degraded more slowly than disordered/α-helix structures. WG foams showed only a small global warming potential and lower fossil carbon emissions than synthetic foams on a mass basis, as illustrated with a nitrile-butadiene rubber (NBR) foam. Unlike NBR, the protein foams could be recycled into films, offering an alternative to immediate composting.

## 1 Introduction

Non-biodegradable polymers, such as traditional plastics derived from fossil-based resources, have an important role in many industrial applications due to e.g., their low density, affordability, high strength, and mechanical flexibility.^1^ However, these materials pollute the environment and persist for centuries, accumulating in landfills, oceans, and natural habitats without natural decomposition.^2^ Biodegradable polymers such as several polyesters, polysaccharides, and proteins can offer a sustainable alternative and have emerged as a promising solution to address environmental challenges in e.g., packaging, agricultural films, and biomedical devices.^3,4^ Wheat gluten (WG) is a biodegradable candidate, which is available as a co-product of wheat starch production.^5,6^ WG can be thermally processed into various products with different properties ranging from stiff foams to flexible films because of its highly cohesive properties and viscoelastic nature, making it a material option for producing foams through conventional polymeric processing techniques, e.g., extrusion.^7−11^ Products have been made with good electric and thermal conductance,^12−14^ high liquid absorbency,^15−18^ and microbial resistance.^19,20^ Furthermore, wheat gluten foams have also demonstrated promising mechanical and structural properties, making them suitable for a variety of applications. In the previous work, the effects of multifunctional additives gallic acid (GA), citric acid (CA), and genipin (GNP) on the mechanical properties and structure integrity of WG foams were investigated, including parameters such as compression strength, energy absorption, and durability under cyclic loading.^21^ In this work we take it further and analyze the end-of-life possible scenarios for these materials, as well as investigating their performance in different harsh environments.

Wheat gluten is a biodegradable material, breaking down by microorganisms, with the specific decomposition products, as well as the degradation rate, depending on the specific environmental conditions (relative humidity, temperature, presence of oxygen, etc.).^22,23^ It has been shown that WG biodegrades in both liquid media and farmland soil^24,25^ and in blends with other biodegradable polymers (e.g., polycaprolactone), as well as in different chemically modified states.^26,27^ However, systematic investigations on the biodegradation and end-life-scenarios of WG remain still largely unexplored, especially when considering the presence of multifunctional additives. Specifically, these additives not only improve the mechanical and functional properties of the foams,^21^ but also influence the environmental effects on WG. For instance, GA provides antimicrobial and antifungal properties, which potentially slow down microbial impact. CA accelerates hydrolytic degradation in alkaline environments, whereas, GNP contributes with cross-links,^21^ increasing structural stability and reducing the degradation rate. For the future replacement of today's plastics in e.g. single-use products (such as sanitary products and packaging) with protein-based plastics, it is important to understand the cradle-to-cradle properties of the latter.

Hence, in this work, the aim was to determine the biodegradation features of glycerol-plasticized WG foams in specific relevant environments, also containing the multifunctional additives. As demonstrated in previous work, the additives improved several foam properties,^21^ and as a continuation, this work investigated biodegradation in water at different pHs (acidic, neutral, and basic), in soil, and in air with a high relative humidity (∼100%). The focus on foams was based on the extended recent studies on the properties of WG-based foams^7,8,13,14,20,21,28,29^ and cradle-to-cradle analysis related to the use of those materials. Besides the biodegradation features, the study considers key end-of-life aspects, including an impact assessment of the global warming potential of the WG foams and a nitrile-butadiene rubber training-mat foam, previously used as a reference for non-biobased and non-biodegradable products. The bioassimilation and fertilizing properties of the foams using fast-growing coriander seeds were also evaluated. As related to biodegradation and soil enrichment/plant growth, the sample moisture uptake, antioxidant and antibacterial properties, and mold resistance were determined. The possibility of using mechanical recycling forming a new WG product, rather than immediate composting, was also demonstrated.

## 2 Material and Methods

### 2.1 Materials

The wheat gluten powder was supplied by Lantmämmen Reppe AB, Lidköping, Sweden. The composition has been described before;^21^ the main component is wheat gluten proteins (85.2 wt %). Glycerol (ACS ≥ 99.5%), ammonium bicarbonate (ABC, NH_4_HCO_3_, ACS Reagent≥ 98%), and gallic acid [GA, ACS Reagent ≥ 97,5% (titration)], were provided by Sigma-Aldrich, Sweden. Chloroform (ACS Reagent ≥ 99%) was supplied by Sigma-Aldrich, Germany. The citric acid (CA, ACS Reagent ≥ 99.5%) and genipin (GNP, ACS Reagent ≥ 98%, HPLC grade) were purchased from Merck Life Science AB, Sweden, and Zhinxin Biotechnology, China, respectively. A nitrile-butadiene rubber training-mat foam (NBR, density: 120 kg/m^3^), was obtained from a hardware store (Jula AB, Sweden). Urea (ACS ≥ 99–100%) was supplied by Fisher Chemical as industrial fertilizer in the tests. Seeds of *Coriandrum sativum* were obtained from Agro RBD, CA, Venezuela; Non-fertilized soil from El Horticultor JJR, C.A, Venezuela; and fertilizer from La Fortaleza C.A, Venezuela.

### 2.2 Foam Preparation

**Figure 1:**
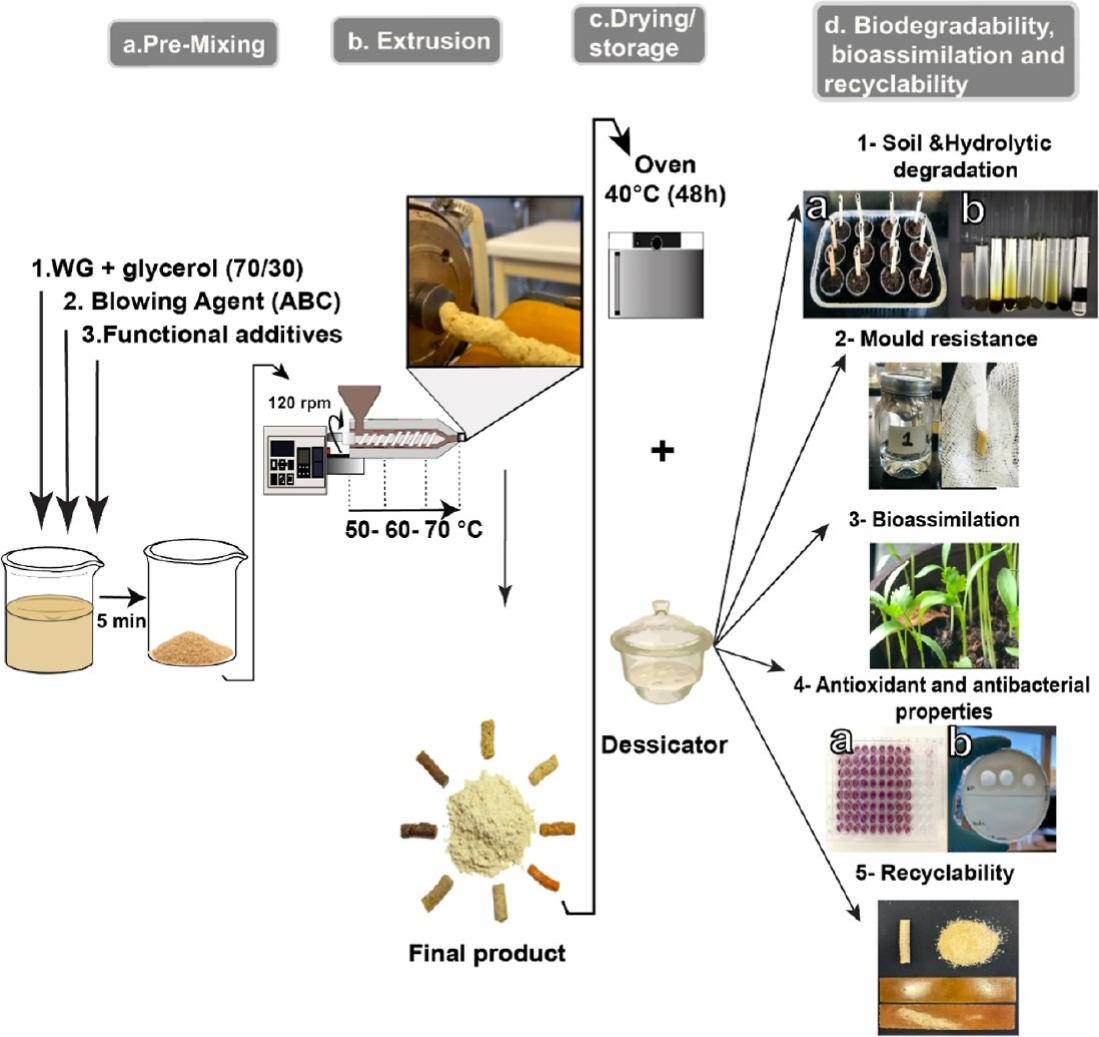
Illustration of biodegradability and bioassimilation assessments of wheat gluten foams using multifunctional additives.

The sample preparation process was the same as in the previous work (Figure 1a,b).^21^ To obtain 50 g of the WG/glycerol mixture (WG/G), 35 g (70 wt %) of WG powder was poured into a beaker containing 15 g (30 wt %) glycerol, and these were then manually mixed for 5 min until a homogeneous WG-glycerol mixture was obtained, with a mass ratio of 7/3 (WG/glycerol). The 30% of glycerol content was selected based on previous work, which demonstrated that this composition resulted in a combination of mechanically flexible and ductile films with good extrudability.^30^ In all but the reference WG/G sample, 5 wt % (based on 100% WG/G) of ammonium bicarbonate (ABC) was added as a blowing agent. 1 and 5 wt % (based on 100% WG/G) gallic acid, citric acid, or genipin were then added to the mixture before the extrusion.

The mixture was extruded in a single screw extruder (Brabender Do-Corder C3) with an L/D ratio of 20 and a screw compression ratio of 2.5. The heating zones were set to 50–60–70 °C from the hopper to the die to build up the ABC foaming reaction (eq 1) gradually toward the die section.^31^ The screw speed was 120 rpm, and a circular die with a diameter of 6.5 mm was used. The weight ratio of the multifunctional additives and the operational processing conditions were chosen based on optimization trials. The extrudates were dried overnight at 40 °C in a ventilated oven and were then stored in a desiccator containing silica gel for at least 1 week before any test [relative humidity (RH) ≤ 10%, Figure 1c]. The full description of the samples is given in Table 1. The reference samples prepared with glycerol and ammonium bicarbonate were named WG/G and WG/G/ABC, respectively, whereas the samples with the GA, CA, and GNP additives were named as e.g. WG/G/ABC/1GA, where the number refers to the added proportion of GA, CA or GNP in percent.
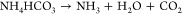
1

**Table 1:** Summary of the WG Foam Compositions and Foam Density^a^

sample	WG^b^	G^b^	ABC^c^	GA^c^	CA^c^	GNP^c^	density^d^	total porosity^e^	open porosity^e^	pore size^f^
WG powder							1290^g^			
WG/G	70	30					883 ± 2^e^	31.6 ± 0.2^b^	1.9 ± 0.1^a^	65 ± 30^a^
WG/G/ABC			5				720 ± 7^b^	44.1 ± 0.6^d^	7.7 ± 0.5^b^	215 ± 159^ab^
WG/G/ABC/1GA				1			840 ± 20^d^	35.0 ± 1.8^c^	16.4 ± 2.6^d^	145 ± 80^ab^
WG/G/ABC/5GA				5			820 ± 20^cd^	36.7 ± 1.8^c^	20.0 ± 2.2^d^	190 ± 100^ab^
WG/G/ABC/1CA					1		641 ± 6^a^	50.2 ± 0.5^f^	10.9 ± 1.4^c^	195 ± 116^ab^
WG/G/ABC/5CA					5		650 ± 3^a^	49.4 ± 0.2^e^	7.5 ± 0.4^b^	183 ± 69^b^
WG/G/ABC/1GNP						1	950 ± 40^f^	26.1 ± 2.9^a^	11.8 ± 3.5^cd^	190 ± 108^ab^
WG/G/ABC/5GNP						5	804 ± 10^c^	37.5 ± 1.1^c^	26.5 ± 1.8^e^	154 ± 55^b^

a Note: Density, porosity and pore size from ref (21). Different letters mean the values are significantly different (*P* < 0.05) in each column.

b (wt %).

c (wt %/100 g WG/G).

d (kg/m^3^).

e (%).

f (μm).

### 2.3 Soil Degradation Test

The WG foam samples were assessed in soil degradation tests according to previous work.^32,33^ Briefly, 1 cm of the extrudate rod was buried at a depth of 1.5 cm in a predrilled plastic cup with formulated composting soil. It consisted of 3 kg basic soil with 25 mL of fertilizer (10% nitrogen, 4% phosphorus, 7% potassium, 0.8% sulfur, and 0.2% magnesium as macronutrients) in 1 L of water. The fertilizer and soil weight ratio was selected based on the manufacturer’s recommendation, which showed that this composition yielded the best fertilization properties.^34,35^ The test was performed at 25 °C. The system was placed in an aluminum tray containing the formulated soil to have a contact base (Figure 1d.1a). To study biodegradation, the samples were removed from each composting system after 1, 2, 3, 5, 7, and 8 weeks until pieces of samples could not be observed in the composting soil. The samples were carefully unearthed from the soil, and excess soil was removed mechanically from the sample, which was subsequently washed with distilled water and dried in an oven at 35–40 °C until a constant weight was obtained by using a U.S solid balance SKU: JFDS00008 (USA) with 0.1 mg reading. The photographed samples’ visual appearance was determined as a function of time to capture the morphological changes during degradation, and the weight loss was determined by calculating the percentage of the weight retained during the exposure to the degradation environment (eq 2).
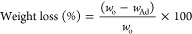
2where *w*_0_ and *w*_Ad_ are the initial weight of the foam and its weight after degradation (including extraction, cleaning, and drying until reaching constant weight), respectively.

### 2.4 Hydrolytic Degradation Test

#### 2.4.1 Buffer Preparation

Buffers were prepared at pH 4, pH 7, and pH 10. The 0.1 M pH 4 buffer solution was produced with acetic acid/sodium acetate (CH_3_COOH and C_2_H_3_NaO_2_) supplied by Sigma-Aldrich (Germany), while 0.1 M pH 7 buffer was obtained using sodium biphosphate/disodium phosphate (NaH_2_PO_4_, and KH_2_PO_4_) from Merk (Germany). The 0.1 M pH 10 buffer was prepared with potassium bicarbonate/potassium carbonate (KHCO_3_ and K_2_CO_3_) supplied from Himedia, India. The pH values of all buffers were measured immediately before use.

#### 2.4.2 Hydrolytic Degradation Test

Hydrolytic degradation experiments were conducted on extruded segments of 0.5 cm of WG and NBR foams (Figure 1d.1b) in line with those in ref (36). The specimen were first dried at 40 °C and then submerged in 10 mL of buffer at pH 4, pH 7, and pH 10 at 25 °C for 5 weeks until sample pieces could not be observed in the buffer solution. At the end of each week, the samples at each pH were removed and dried at 35–40 °C until a constant weight was obtained. The pH of the remaining solutions was measured immediately after the removal of the samples. The pH of the buffer solution and the weight loss were recorded by using a pH-meter inlab expert Pro-ISM (Mettler Toledo) and VWR Ioniser balance (UK) (with a reading of 0.1 mg), respectively. The weight loss was calculated according to eq 2.

To determine changes in molecular weight of the WG samples during hydrolytic degradation, an SDS–PAGE test (Bio-Rad Mini Protein TGX Precast vertical electrophoresis cell, Sweden) was used. Ten μL solution of degraded samples was mixed with an equal volume of a Sigma Laemmli 2× concentrate buffer obtained from Sigma-Aldrich, Sweden, and an Invitrogen SeeBlue Plus 2 prestained protein, used as standard obtained from Thermo Fisher Scientific, Sweden. The samples in the electrophoretic trays were carried out at a voltage of 200 V for 20 min, and the images were obtained from the GelDoc Go molecular biology bundle.

### 2.5 Fourier-Transform Infrared Spectroscopy

The samples biodegraded in soil were analyzed with a Bruker Tensor 27 FTIR (Germany), equipped with a single-reflection ATR having a ZnSe crystal. The scanning step was 1.0 cm^–1^ with a resolution of 4.0 cm^–1^. The final spectrum was based on 64 consecutive scans from 4000 to 500 cm^–1^. In contrast, the FTIR spectra of the samples biodegraded in hydrolytic conditions were obtained with a PerkinElmer Spectrum 100 (USA), equipped with a triglycine sulfate (TGS) detector, and a Golden Gate unit (Single-reflection ATR, Graseby Specac, England). The scanning step was 1.0 cm^–1^ with a resolution of 4.0 cm^–1^. Sixteen consecutive scans were recorded (4000–600 cm^–1^) for each sample.

### 2.6 Scanning Electron Microscopy

SEM was performed using a Field emission SEM (JEOL JSM-6390, Japan) at a voltage of 30 kV The foamed samples were frozen by immersing them in liquid nitrogen for 30 s and then broken into pieces. These were fixed onto aluminum specimen holders using conductive carbon tape. The samples were coated with gold, using a sputter coater (model SCD-030, Japan) for 10 min.

### 2.7 Mold Resistance and Water Uptake Test

The mold growth and water uptake test were performed by using a 1 cm piece of the extrudate in an airtight glass container (Figure 1d.2). The sample was placed on a sterile gauze placed between the cap of the glass container and 20 mL distilled water in the bottom, to obtain ∼100% relative humidity (RH). The container was closed with the cap and covered with aluminum foil to prevent any entry or exit of moisture and exposure to UV-light. The weights of the samples were measured at regular 24 h intervals for 6 days using the U.S. analytical balance. Before the test was performed, the samples were dried in an oven at 35–40 °C until a constant weight was obtained. The percentage of water uptake was calculated according to eq 3
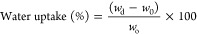
3where *w*_d_ and *w*_0_ are the weight after exposure at 100% moisture and the initial weight of the foam, respectively.

### 2.8 Bioassimilation Test

A representative study on the impact of the biofoams simulating disposal of WG foams and NBR in the environment was assessed using fast-growing seeds of *Coriandrum. sativum*, *coriander* (Figure 1d.3). 3.5 g of fragments of the selected samples were buried in cylindrical plastic containers (diameter 10 cm, height 10 cm), following the sowing procedure described in ref (37), which consists of using three principal layers: a bottom layer of soil mixed with WG sample material, a middle layer of coriander seeds and finally an upper layer of soil mixed with WG sample material, thus making sure that the coriander seeds were surrounded by WG containing soil. The coriander seeds were evenly dispersed up to a density of 40 seeds (0.5 g)/100 cm^2^ according to the recommendation from the manufacturer.^38^ The basic soil was without any added nutrition (5.0 kg of soil with 2.0 L of water to obtain moist soil). The irrigation system was the same for each formulation [25 mL of water, added three times per week (pH ∼ 6)].^39^ NBR synthetic samples, standard soil, fertilized soil (25 mL of liquid fertilizer/L water for each, combined with 3 kg of soil), and urea in water (0.5–2 wt %) were separately used as controls using the same procedure as before, only with the difference that these were used instead of the WG foam. Germination rates and plant morphologies, such as leaf diameter and total height, were measured to analyze the fertilization effect of the biobased foams. The data were collected after 10, 20, and 30 days from the sowing.

Leaf chlorophyll content is an important indicator of leaf greenness and is commonly used to assess nutrient deficiencies and monitor changes in plant health. To analyze the chlorophyll content in coriander leaves, a pigment extraction procedure was implemented based on a methodology used in ref (40). 0.5 g of coriander leaves from the samples were ground in 7 mL of chloroform until a homogeneous mixture was obtained. 300 g of the mixture was centrifuged in a Digisyste model DSC-158T centrifuge (Taiwan) for 20 min to separate the solid phase from the aqueous phase. The resulting solution was immediately transferred to an amber glass container to protect it from UV light, and it was diluted with 7 mL of chloroform to obtain the same volumetric concentration in all samples. The absorption spectra of the solutions were recorded using a UV–visible spectrophotometer (Agilent 8453, USA) at 450–480 nm and 640–670 nm to determine chlorophyll A and B, respectively. The total chlorophyll content (CC) was obtained using a calibration curve, in accordance with the procedure of Mackinney et al.^41^ and Kirk and Allen^42^ using eq 4
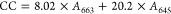
4where *A*_i_ corresponds to the UV absorbance value at wavelength *i*.

### 2.9 Antioxidant and Antibacterial Assessment

The antioxidant activity was assessed by analyzing the scavenging activity against the radical DPPH (1,1-diphenyl-2-picrylhydrazyl) (Figure 1d.4a). Following the method used in previous work,^43^ the radical scavenging activity of WG-based porous materials was determined as the percentage of DPPH remaining in the solution after three cycles of oxidation to determine whether the antioxidant properties could be maintained over time. Microplates were read in a microplate reader ClarioStar (BMG Labtech, Germany) using the absorbance at 517 nm. The radical scavenging activity from individual additives used in the manufacturing of WG foams was determined by mixing DPPH (0.2 mM methanolic solution) with different volumes of an aqueous/methanol solution containing the additive in concentrations between 100 down to 0.5 mg mL^–1^ and then leaving the mixture in the dark for 30 min. The results were expressed as EC50, representing the concentration of antioxidants required to reduce the initial concentration of DPPH by 50%.

The antibacterial activity of the wheat gluten foams was assessed with the disk diffusion assay method (Figure 1d.4b). The bacteria *Escherichia coli* (CCUG 10979, *E. coli*) and *Bacillus cereus* (CCUG 7414, *B. cereus*) were used to represent the effects of potent skin pathogen bacteria of either Gram-negative (*E. coli*) or Gram-positive pathogen (*B. cereus*). *E. coli* strain was inoculated in TSB (Tryptic Soy Broth) medium, while *B. cereus* was inoculated in an LB (Lysogeny Broth) medium. The cell density was adjusted on the McFarland scale of 0.5 with the aid of a spectrophotometer. The inoculated media (100 μL) was coated on the surface of solidified agar plates, whereafter samples were placed on the dried surface of the plates. The antibacterial activity was evaluated qualitatively by the observation of any bacterial inhibition zone and of bacterial growth on the surface of the material after incubation at 37 °C for 24 h.

### 2.10 Recycling Properties

Compression molding was performed in a platen hot press (Fontijne TP-400, The Netherlands) to determine the recyclability of WG foams (Figure 1d.5). The WG foam was ground at 6000 rpm using a Retsch Ultra Centrifugal Mill ZM 200 (Germany) equipped with a ring sieve labeled 1 before pressing. The sample was hot pressed into 1 mm thick rectangular molds with sides 120 and 30 mm. Polytetrafluoroethylene (PTFE) sheets were placed on both sides of the sample to prevent the material from sticking to the metal plates during the pressing, and 10 mm thick plates were placed between the press plates and the PTFE sheets to distribute the pressure more even over the mold. Ca. 4.5 g of the gluten mixture was placed evenly into the mold and was then compacted at room temperature using a 250 kN press force for 1 min to exhaust air. After the pressure was released and the press plates of the hot press were separated, the temperature was raised to 130 °C, and the samples were pressed again using a force of 250 kN for 15 min. Subsequently, the samples were cooled to room temperature while remaining under pressure and then removed from the press. A new (non-recycled) WG mixture was separately prepared as a reference. For a 50 g batch of WG/G, 35 g (70 wt %) of WG powder was manually mixed with 15 g (30 wt %) of glycerol until a homogeneous mixture was obtained. When using the foaming agent and multifunctional additive, 2.5 g (5 wt %) of ABC and CA were added during the mixing. The mixture was hot-pressed using the same procedure as above. The new samples prepared with glycerol and citric acid were named WG/G-NR and WG/G/ABC/5CA-NR, respectively, whereas the WG foams, ground, and then reshaped into films were named WG/G-R and WG/G/ABC/5CA-R.

Tensile testing of the samples was performed at 23 °C and 50% RH using an Instron 5944 universal testing machine (USA) equipped with a load cell of 500 N. The extension rate was 10 mm/min, and five dumbbell-shaped specimens of each sample were used. The stress–strain curve of each specimen was measured, and the elastic modulus (*E*) was calculated from the slope of the linear region (below 5% strain). The tensile stress (σ_b_) and elongation at break (ε_b_) were taken as the maximum stress value and the elongation at the last recorded data point before failure, respectively. The toughness (*U*) was calculated as the area under the tensile curve. The specimens were conditioned at 50% RH and 23 °C for at least 72 h prior to the tensile testing.

To evaluate protein structural changes, the samples’ FTIR spectra were recorded with the PerkinElmer spectrum 100 machine using the same procedure as described above.

### 2.11 Green-House Gas Emission Assessment

The greenhouse gas (GHG) emissions from the production and end-of-life carbon release of the plasticized WG-based foams and reference material (for the NBR training mat, the components used were taken from ref (44)) were estimated. The assessment was carried out based on the components used in production (Tables 1 and S1) and corresponding emission data (Table S2). The differences between the production processes of the plasticized WG-based foams and the reference material were assumed to be negligible, and the impact of the processing was therefore excluded from the assessment.

### 2.12 Statistical Analysis

Statistical analyses were performed with the least significant difference (LSD) in Fisher’s procedure, evaluating the significance of the measurements (*p* < 0.05, 95% confidence level). These analyses were performed with the software Statgraphics 18 (USA). At least triplicates were used in each measurement.

## 3 Results and Discussion

### 3.1 Soil Degradation

#### 3.1.1 Weight Loss, Visual Observation and Odor

**Figure 2:**
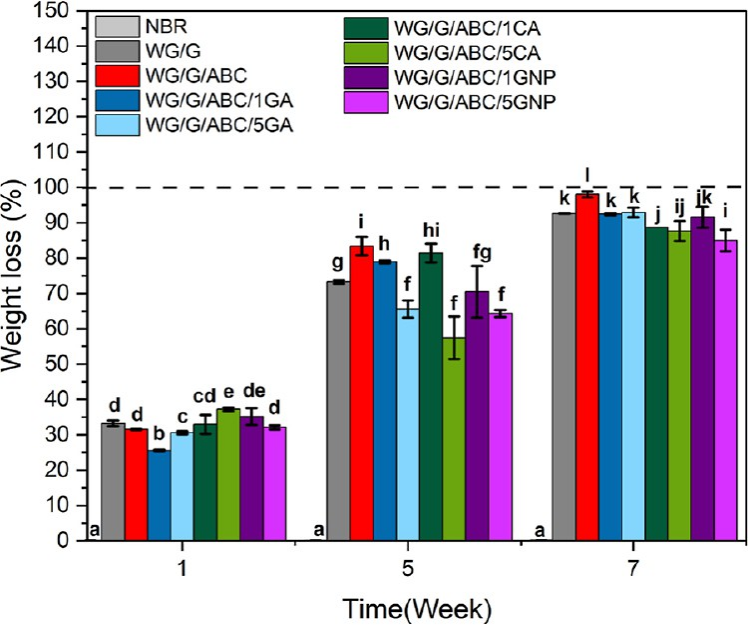
Weight loss of the foams after 7 weeks during biodegradation in soil. Note: Different letters mean the values are significantly different (*P* < 0.05).

Figures 2 and S1 show the weight loss of the different samples after 1–8 weeks (2, 3, and 8 weeks in Figure S1) in the soil. The weight loss after 1 week was mainly due to the loss of glycerol, but also possibly the loss of some additives and species from the WG raw material into the soil and during the cleaning, washing, and drying processes.^45,46^ After the second week, the rate of degradation increased considerably in all WG foams, leading to an increase in weight loss of up to 50% after 5 weeks (Figures 2 and S1). The WG/G/ABC foam underwent the largest weight loss (85% in 5 weeks), followed by the foam with high citric acid content (WG/G/ABC/5CA, 82%) (Figure 2). After 8 weeks, the foams with 1 and 5 wt % genipin had lost the least material, whereas the loss was similar for all the other WG foams (Figure S1).

The high degree of degradation of the WG foam sample can be attributed partly to the high content of nitrogen. Nitrogen is one of the bases of nutrition of plants and microorganisms that can be found inorganically as NH_4_^+^ or NO_3_^–^ or organically from protein peptide bonds that are considered the predominant source of organic nitrogen in soils.^47,48^ In this environment, bacteria, such as *Actinomycetes* perform the critical role of degrading materials such as proteins by breaking peptide bonds.

Figure S2a displays the visual appearance of all foams before being placed in the soil and after 7 weeks in it. During the degradation, the samples showed notable morphological changes and foam resilience decreased (mainly due to early loss of glycerol), leading to erosion, cracking, and embrittlement within the first few weeks. Changes in color from beige to black, a structural collapse, and apparent soil fauna (presence of larvae and worms) were also observed from 3 weeks and onward (Figure S3 and Video S1).

During the degradation process, odors from the foam decomposition products were perceived, ranging from sweet and sour smells of decomposed fruits to feces smell, depending on the sample formulation. These odors are generated possibly from the production of esters and alcohols formed during the fermentation of sugars of organic material, as well as the formation of nitrogen-containing species and carboxylic acids. Hydrogen sulfide (H_2_S) is also formed due to the presence of methionine and cysteine in the protein.^49−52^

The degradation occurred, in general, faster in the interior of the rod foams, as observed after 3 weeks (Figure S2b). This is because the WG foam outer regions were often denser and less accessible for the microbes than the interior, a consequence of the pressure exerted by the barrel wall on the material during the foam extrusion process.^21^ By comparing the initial rate of soil degradation (weight loss after the first and second weeks, when the structure of the foams were still resembling the pristine foam, Figures 2 and S2b) with the foam structure (total/open porosity and pore size, Table 1) of the pristine foams, no correlation between these was observed. Finally, it should be mentioned that, as expected, the NBR did not show any degradation during the 8 weeks.

The formation of mold on the sample surfaces was another indicator of microbial activity in the soil samples (Figures S4 and S5). These fungal micelles appeared in the WG samples already after 1 week, covering 80% of the surface, and 100% coverage was observed after 2 weeks. Morphologically, this proliferation probably corresponds to fungi of the *Trichoderma*, *Penicillium*, and *Aspergillus* families, according to previous reports.^53−56^ The presence of these organisms is due to the spores normally present in the air, and the soil proliferates.^57,58^ Notice that citric acid is one of the main organic acids produced in fungal fermentation, not the least from *Aspergillus niger*, and it is the key intermediary in the Krebs cycle. This feature, as well as the relatively low density (650 kg/m^3^) and high total porosity (∼50%) of the 5 wt % citric acid foam (Table 1) facilitated its bioassimilation and high microbial activity.^59,60^ As expected, no mold growth was observed on the NBR foam (Figures S2a and S5a).

#### 3.1.2 FTIR and Protein Secondary Structure

**Figure 3:**
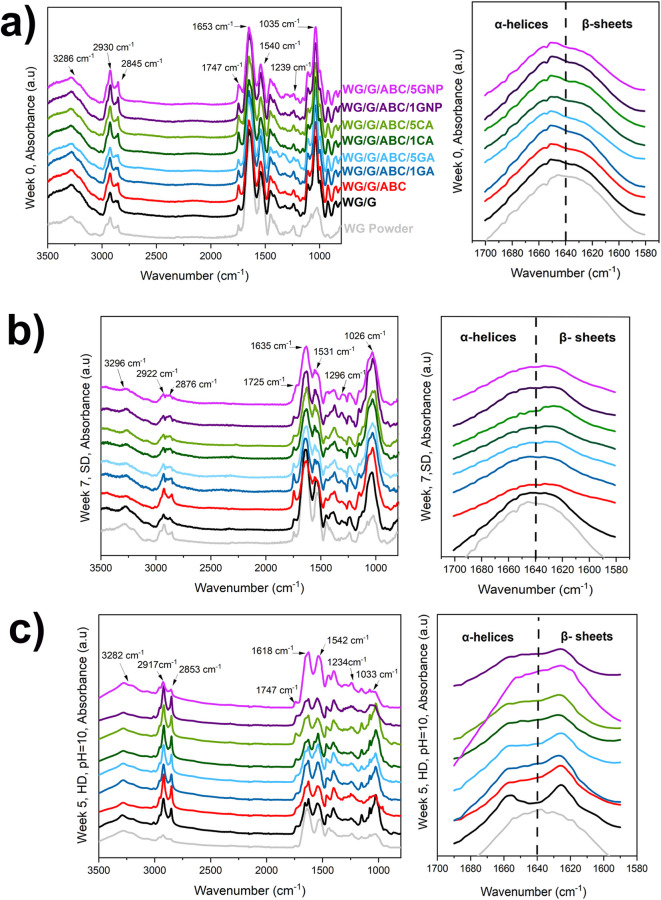
Full FTIR spectra and the amide I region (1700–1580 cm^–1^) of the samples in different environments: (a) before degradation, (b) soil degradation (SD) after 7 weeks, and (c) hydrolytic degradation (HD) at pH 10 after 5 weeks.

The full FTIR spectra of the samples before and after degradation in soil are shown in Figure 3a,b. The undegraded WG foams showed a broad and intense band at 3286 cm^–1^ assigned to O–H and N–H vibrational stretching, originating from WG, glycerol, and the additives (Figure 3a).^61,62^ The bands at 2930 and 2845 cm^–1^ originated from aliphatic C–H bond vibrations.^63^ In the region 1800–1200 cm^–1^, peaks were present at 1653, 1540, and 1239 cm^–1^, corresponding to –C=O stretching (Amide I), the bending of the N–H bond (amide II), and the C–N stretching and N–H bending (amide III), respectively. Furthermore, in the region 800–1150 cm^–1^, bands originating from vibrations associated with C–C and C–O bonds/O–H deformation in glycerol were observed, with bands at ∼850, 920, 995, 1035, and 1104 cm^–1^.^64−66^ All foams showed a change in the absorbance profile in the amide I region (1650–1580 cm^–1^) compared to that of the pure wheat gluten powder, indicating a change in the secondary molecular structure during the manufacturing.^21^

In line with the weight-loss data during the soil degradation test, the size of the bands in the 3700–3000 cm^–1^ region decreased from week 0–7, showing the loss of nitrogen-containing species (such as ammonia and amines) and hydroxyl/carboxyl species (Figure 3a,b). Moreover, the peaks around 2922 and 2876 cm^–1^ decreased slightly after week 7. This was partly due to the loss of glycerol and also the loss of degradation products from the aliphatic hydrocarbon parts of the protein chains. Interestingly, a decrease in the 1725 cm^–1^ band after week 7 was observed, probably a consequence of the degradation products containing the functional group C=O, such as carboxylic acids, aldehydes, and/or ketones.

Changes in the secondary structure of the protein occurred during the soil degradation test, as observed by the changes in shape and size of the amide I/II and III regions: 1635–1531 and ∼1296 cm^–1^, respectively. An interesting feature, never reported before, was observed in the amide I region (1700–1580 cm^–1^); the curve shape for all WG foams (peaking in the 1700–1640 cm^–1^ region) indicated a sizable amount of α-helices and random coil (unordered) protein chain segments in the undegraded material. However, after 7 weeks of degradation, the curves peaked in the 1640–1580 cm^–1^ region, indicating a higher relative content of β-sheets in the aged material. The denser and more energetically stable β-sheet structure did not seem to be as accessible to the microorganisms and enzymes as the more open α-helix/random coil structure.^67^ It should be mentioned that both glycerol and water have bands in the amide I region. However, the loss of glycerol or the uptake of water would not yield the observed changes.^68,69^ Finally, the peaks in the 1200–1000 cm^–1^ region showed significant variations, attributed to C–O and C–C stretching in different structures. These changes suggest that the degradation process also involved carbohydrates and other oxygen-containing components in the WG raw material.

Figure S6 shows the full FTIR spectra of the NBR foam. The band at 3521 cm^–1^ is ascribed to the O–H stretch.^70^ Bands at 2845 and 2231 cm^–1^ originate from, respectively, the CH_2_ group and the CN group.^71^ The carbonyl group C=O contributes to the formation of a peak at 1727 cm^–1^, possibly due to the presence of lubricant/plasticizer from the rubber processing since this band does not correspond to the base structure of NBR. The peak at 1596 cm^–1^ is associated with the –C=C bond.^70,72^ Finally, the band at 978 cm^–1^ is related to the −CH=CH– (trans) bond of the butadiene component. In accordance with weight loss data, no significant variations in the NBR FTIR spectrum was observed in the soil degradation test.

#### 3.1.3 Observations by Scanning Electron Microscopy

**Figure 4:**
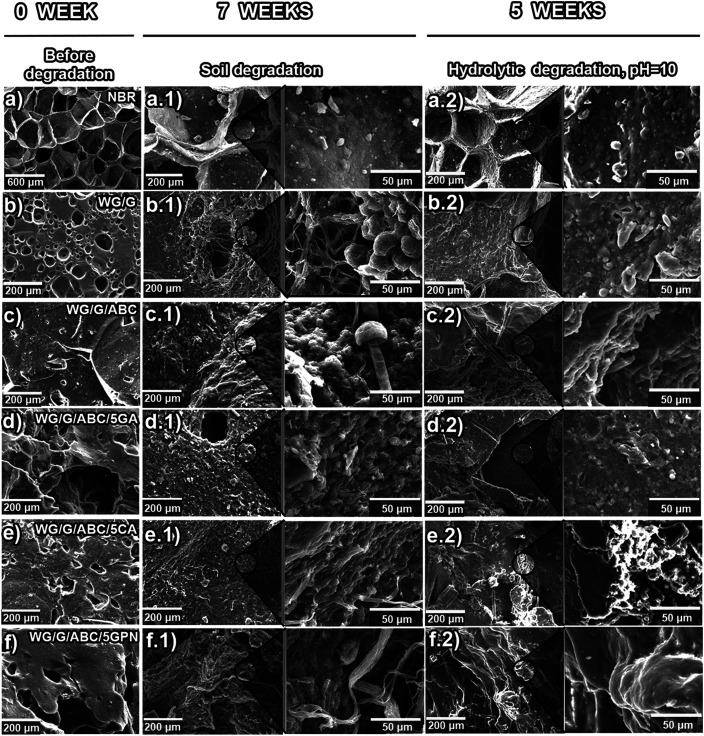
Cross- sections revealed by SEM of extruded samples after 7 weeks in soil and after 5 weeks in alkaline aqueous solution.

Figures 4 and S7 show the morphology of the WG foams before and after soil degradation. After soil degradation for 7 weeks, several structural changes were observed compared to the starting materials, involving denser and eroded structures in the former, which were in line with greater weight losses at longer degradation times (Figures 2, 4b.1–f.1), S1 and S7b.1–f.1. Furthermore, at high magnification, the presence of microbial activity was discernible [(Figures 4b1–f.1) and S7(b.1–f.1)]. In the WG/G sample, bacteria with an elliptical shape was observed, possibly an *Alicyclobacillus Acidoterrestris*-type, as previously reported in ref (73) (Figure 4b.1). At the surface of the WG/G/ABC/1CA and WG/G/ABC/5CA samples, long threads or filaments/microtubules of possibly *Paecilomyces varioti* appeared (Figures 4e.1 and S7e.1).^74^ As expected, no microbial activity was observed on the NBR foam surface (Figure 4a.1).

### 3.2 Hydrolytic Degradation

#### 3.2.1 Weight Loss and Visual Observations

**Figure 5:**
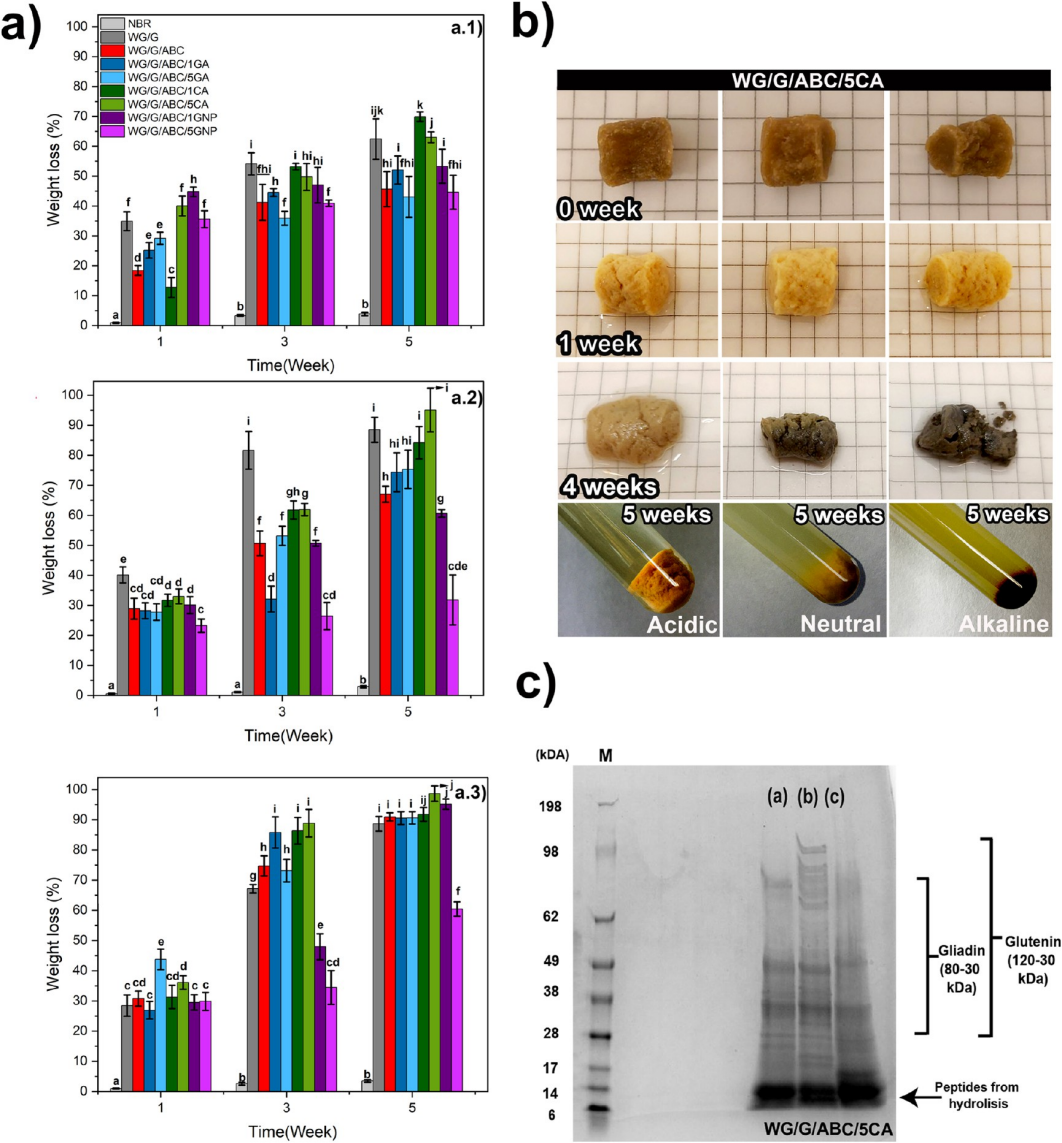
(a) Weight loss after weeks 1, 3, and 5 in acidic (a.1), neutral (a.2), and alkaline (a.3) conditions, (b) The appearance of WG/G/ABC/5CA during the first 4 weeks, (c) electropherogram of WG/G/ABC/5CA, after 4 weeks in (a) acidic, (b) neutral, (c) alkaline conditions, showing the banding pattern and molecular weight marker. Note: Different letters indicate that the values are significantly different (*P* < 0.05).

Figures 5 and S8 show the results of the hydrolytic degradation of the foams in acidic, neutral, and alkaline conditions. All WG foams showed a large weight loss after 1 week, corresponding to the loss of glycerol and water-soluble additives and WG species (Figure 5a.1–a.3).^7,21,75,76^ After this initial loss, further loss was due mainly to a hydrolytic attack on the solid material. Notably, the highest overall weight loss during the 5 week degradation period was observed in alkaline conditions (Figure 5a.3). In contrast, the lowest overall weight loss was observed in the acidic medium (Figure 5a.1). The largest weight loss (almost 100% in alkaline conditions) was observed consistently for the foam with 5 wt % citric acid (WG/G/ABC/5CA), whereas the foam with 5 wt % genipin (WG/G/ABC/5GNP) showed overall the lowest hydrolytic degradation after 5 weeks (Figure 5a.3). In fact, it showed only a 60% weight loss in the alkaline condition. This indicated the presence of a genipin-based cross-linked structure that experienced essentially only the loss of glycerol at high pH.^21,77^ Moreover, it is well-known that the further the medium is from the protein isoelectric point (PI) (WG ∼ 6.2),^78,79^ the lower is the degree of protein–protein interactions, giving rise to a higher degree of water–protein interactions facilitating the hydrolysis of the protein material.^22,23,80^ The differences in the effects of the different pH on the degradation were also apparent by comparing the appearance of the 5 wt % CA foam in the different media (Figure 5b). The foam became darker, and the geometry of the sample became more irregular from neutral to alkaline conditions (after 4 weeks). On the other hand, the sample in the acidic condition retained both its color and shape better than that in the alkaline condition. Notable erosion was also observed in the 5 wt % CA foams in alkaline conditions (Figure 5b, week 4). On the contrary, the NBR reference showed only a very small mass loss after 5 weeks, without any pH dependence (Figures 5a and S8). It is known that NBR is less susceptible to hydrolysis due to the hydrophobic butadiene part and the sulfur cross-links.^81^

The results also revealed that hydrolytic degradation occurred faster than degradation in soil, with, as mentioned before, the highest weight-loss rate in alkaline conditions, showing the materiaĺs susceptibility to hydrolysis at high pH (Figures 2, 5a, S1, S8 and Table S3 summarizes the weight loss in both the hydrolytic and soil environments). Notably the extent of degradation reached 98% for the 5 wt % CA foam, compared to only 57% for the same sample in soil over the same period. The hydrolytic degradation of WG-based foams involves water uptake and diffusion into the material followed by hydration and scission of intermolecular hydrogen bonds, swelling, and finally, hydrolysis of covalent bonds.^82,83^ In contrast, degradation in soil involves a combination of physical, chemical, and biological processes. All these mechanisms are influenced by soil composition, pH, moisture content, and microbial and enzymatic activity.^84,85^ Naturally, the actual temperature is another important factor in both cases.

#### 3.2.2 pH Evolution and Protein Molecular Weight

Figure S9a shows the pH values determined during the degradation period. The pH values in the initial alkaline system decreased noticeably after 3–5 weeks for all but the 5 wt % genipin and NBR samples, while in the acidic and neutral systems, the pH remained relatively constant. These results indicate that the hydrolysis in alkaline conditions was more prominent, with also larger weight losses, as shown in Figure 5a.3. The reason is the formation of acidic degradation products, such as carboxylic acids (Figure S9b).^45,86,87^ Additionally, the electropherogram of the WG foams indicated that the samples exposed to alkaline conditions exhibited a higher degree of cleavage of peptide bonds yielding shorter peptides (6–50 kDa) than those formed in acidic and neutral conditions (6–100 kDa) (Figures 5c and S10).

It should be noted that in future work, it is of importance to take he investigation further and explore the biodegradation features in field-trails where the conditions are less controlled, involving also several types of habitats.

#### 3.2.3 FTIR and Protein Secondary Structure

The full FTIR spectra of the WG foams recorded during hydrolytic degradation indicated considerable differences compared to the unexposed material (Figures 3c and S11b,c). A substantial loss of glycerol was observed by the significant decrease in the 1033 cm^–1^ band intensity (compared with the soil degradation FTIR data in Figure 3b). The size of the bands in the amide I (around 1618 cm^–1^) and the amide II (around 1542 cm^–1^) regions decreased in several of the systems relative to the aliphatic C–H stretch bands (2853, 2917 cm^–1^) (Figures 3c and S11b,c). Moreover, the results indicated a decrease in the amount of peptide bonds (due to peptide chain scissions) at pH4 and pH10 during the hydrolytic degradation, with an increase in the β-sheet content relative to α-helix and random coil content. The latter observation indicates that the hydrolysis is slower in the more dense and energetically stable β-sheet structures (Figures 3c and S11b). However, at pH 7, this effect was less pronounced due to the buffer solution system being close to the isoelectric point with an overall more compact (less denatured) protein, consequently resulting in less protein–water interactions and slower hydrolysis (Figure S11c).^78^ As expected, the NBR FTIR spectrum showed no significant variations in molecular structure in the hydrolytic degradation test (Figure S6).

#### 3.2.4 Scanning Electron Microscopy

Figures 4 and S12 show the morphology of the degraded samples after 5 weeks of immersion in acidic, neutral, and basic aqueous solutions. For the samples subjected to an acidic medium, it was observed that the surface was slightly less rough compared to that at neutral pH due to possible surface erosion of the material (Figure S12b.1–f.1). The erosion was more extensive for the specimens exposed to alkaline conditions, and microcracks appeared (Figure 4b.2–f.2), in line with the higher degradation rates in this medium (Figure 5a.3). For the 5 wt % GNP extrudates, no significant microstructural changes were observed during the 5 weeks test in acidic and neutral conditions (Figure S12f.1,f.2), also in line with the low mass losses (Figure 5a.1,a.2). The presence of microorganisms was not observed in any of the foams exposed to hydrolytic degradation, in contrast to those degraded in soil (Figures 4b.1–f.1,b.2–f.2, S7b.1–f.1, S12b.1–f.1,b.2–f.2). The microstructure of the NBR foam showed no signs of structural degradation and, as expected, microbial activity (Figures 4a.2 and S12a.1,a.2). Finally, as with the soil degradation, the initial rate of the hydrolytic degradation (weight loss within the first 2 weeks) of the different samples (Figures 5a and S8) was compared with their initial foam structure (Table 1). Neither a larger pore size, nor an increasing total or open porosity yielded an increase in the degradation rate. Hence, the hydrolytic, as well as the soil, degradation rate was primarily governed by the chemistry and molecular structure of the foams.

### 3.3 Exposure to High Relative Humidity

**Figure 6:**
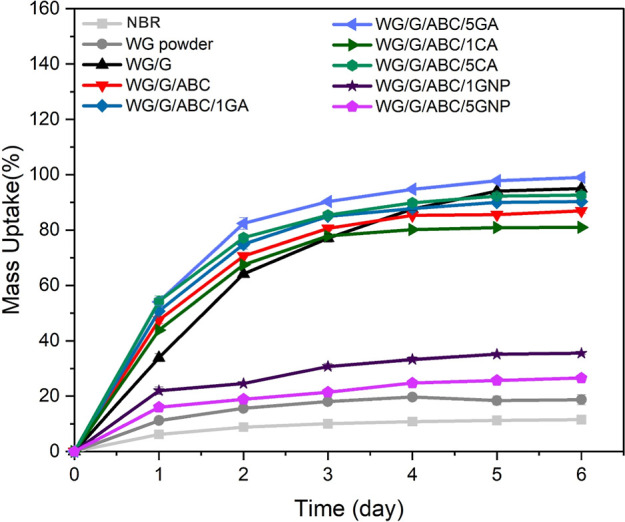
Mass uptake versus time of the foams during high exposure relative humidity.

Figure 6 shows the water uptake in the samples during 6 days at 100% relative humidity. The highest final uptake (ca. 100%) was observed for the foam containing 5 wt % gallic acid (WG/G/ABC/5GA), possibly due to a combination of its low degree of cross-linking, open cell structure, and the high polarity of gallic acid.^21^ The lowest uptake was observed for the NBR samples, followed by WG/G/ABC/5GNP and WG/G/ABC/1GNP (Figure 6). A fast uptake but low saturation uptake was observed for the genipin samples due to its high polarity and the cross-linked structure;^18^ the higher cross-link density is the reason for the lower water uptake in the sample with higher genipin content.^21^ Overall, the size of the open and total porosity, or the pore size, did not determine the size of the saturation moisture uptake (compare the values in Figure 6 with those in Table 1). Hence, the chemistry and molecular structure of the foams had a larger effect on the uptake than the actual foam structure.

Mold was present in all WG foams within the 6 days of exposure, except for the sample with the highest amount of gallic acid (WG/G/5ABC/5GA), due to its antifungal properties (Figure S13). This shows that gluten in combination with glycerol offers a fertile medium for rapid spontaneous fungal growth.^88,89^ The presence of these microorganisms reveals that regardless of the system used here (except that of a high gallic acid content), wheat gluten/glycerol provides the conditions necessary for developing fungal life in a humid environment (RH ∼ 100%). In contrast, the NBR foam and WG powder samples showed high mold resistance (Figure S13). Again, for NBR, this was expected.^90−92^ The WG powder showed no mold growth after 6 days, possibly due to the absence of glycerol.^91,92^ Hence, by choosing different plasticizers, the WG mold resistance can be tailored, at least delayed up to 6 days (the end of the experiment).

### 3.4 Bioassimilation Properties of the Foams

Bioassimilation contributes to an efficient circular bioeconomy and sustainable use of resources. Figure S14 shows the bioassimilation properties of WG foams with the use of fast-growing coriander seeds. The coriander cultivation showed notable compatibility with the WG foams during their cultivation, with germination rates (GR) of up to 97% after 30 days (Figure S15c shows the bioassimilation properties of WG foams with the use of fast-growing coriander seeds. The coriander cultivation showed notable compatibility with the WG foams during their cultivation, with germination rates (GR) of up to 97% after 30 days (Figure S15c). Overall, three growth stages were observed, as reported in refs (93 and 94). All treatments showed an initial fast germination stage (between 3 to 10 days after cultivation), where the seed absorbed water, swelled, and began to develop the radicle (first root) and the cotyledons (the first leaves) (Figure S14a.II). The second stage corresponds to vegetative growth (from days 12–20), where the physiological structure of the plant develops (height and the number of roots, leaves, and branches increase) (Figure S14a.III). Finally, a third stage was observed (from days 20–30), ascribed to the flowering and fruiting stage of coriander, where the plant reaches its maturity and can reproduce (Figure S14a.IV). No exclusion zone for growing seeds was observed around the buried foams, including in the control samples (soil, soil with fertilizer, urea, and NBR) (Figure S16).

During the germination process, the WG material exhibited excellent fertilization properties, especially with the use of biobased foams with multifunctional additives (GA, CA, and GNP) (Figure S15). The leaf diameter, plant total height, and germination rate were overall higher when the WG foams with multifunctional additives were used (GR ∼ 100% in 30 days). In fact, the rate was overall higher than (or similar to) when conventional fertilizers and reference materials were used (Figures S14b and S15a–c).^95^ Hence, the presence of WG protein foams favored the biological activity important for plant growth.^96^ Nitrogen is essential in the formation of amino acids and chlorophyll and provides for healthier leaves, flowers, and stalks. Plants can also absorb nitrogen directly from organic molecules, such as amino acids and peptides, through the model organism in plants called *Arabidopsis thaliana*, where most of the transporters and associated genes have been identified.^97,98^ Additionally, gallic acid has demonstrated a positive effect on plants due to its antioxidant and antimicrobial properties, protecting plants against oxidative stress, defending them from certain pathogens, and promoting root growth, which contributes to overall good plant health and nutrient uptake.^88,89,99^ Moreover, citric acid contributes to antioxidant properties (although observed here only at the low content), improving plant growth and photosynthesis in plant cultivation.^100−102^ Genipin is a nontoxic compound known for its ability to cross-link proteins, and it is used in biomedical and biotechnical products.^103−106^ Genipin has been reported to have antibacterial, anti-inflammatory, and antioxidant properties,^107^ besides its ability to cross-link proteins. It is, therefore, used in biomedical and biotechnical products.^103−106^ The mechanisms for its specific effects and biocompatibility with plants remain to be determined, but based on its natural origin, it shows promise for applications in plant science. Furthermore, as shown in Figure S15c, the reference system where plain soil without fertilizers was used exhibited similar plant morphology/geometry and germination rate up to 30 days as the system with NBR material (GR ∼ 70%).

**Figure 7:**
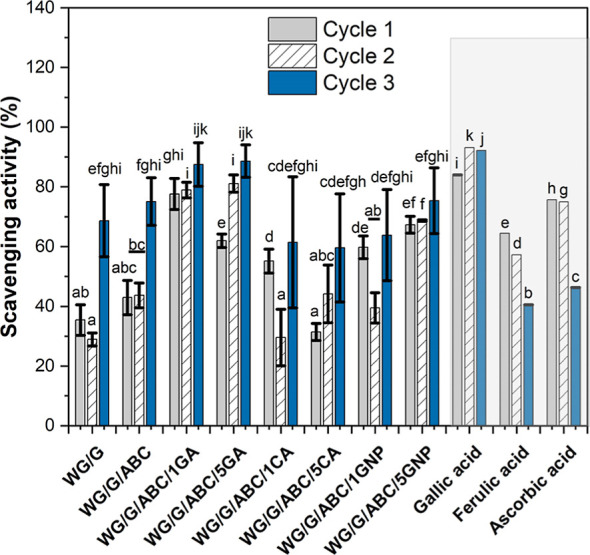
Scavenging activity (DPPH radical inhibition) of WG-based foams and representative species with known antioxidant properties. Note: Different letters mean the values are significantly different (*P* < 0.05).

The addition of 0.5 wt % urea (a commercial fertilizer) had a significant impact on plant growth, with a germination rate of 90% after 30 days. This is attributed to its high nitrogen content.^95^ However, the higher amount of urea (1 and 2 wt %) led to lower GR (70 and 50%), indicating excessive nitrification and a pH change in the soil. Nitrification is a key biological process in the soil nitrogen cycle vital for plant growth whose process occurs in two main stages: ammonification (the decomposition of the protein and organic remains of the plant, releasing ammonium (NH_4_^+^) into the soil by Clostridium ammonifying bacteria) and, nitrification (transformation of ammonium into nitrite (NO_2_^–^) and subsequently nitrate (NO_3_^–^) by oxidizing bacteria, such as nitrosomes and nitrobacter, respectively).^108^ The excessive nitrogen in these two systems indicates possible nitrate toxicity (the leaching of nitrates in the soil), possibly interfering with the absorption of other essential nutrients during plant growth. The results revealed that the use of WG-based materials contributes positively to the optimal morphological growth of plants and demonstrates no toxic effects when disposed of in the environment. Additionally, it was observed in the UV spectra that all the samples contained chlorophyll A (640–670 nm) and chlorophyll B (450–480 nm) pigments (Figure S17).^109^ WG/G, WG/G/ABC, and foams with the multifunctional additives yielded a higher natural pigment content (chlorophyll, CHL) after 20 days of germination compared to the control samples (Figure S18), except for WG/G/ABC/5CA, which shows values similar to the urea 0.5% soil, and the fertilized soil (soil + F). Recent studies have shown that citric acid may be beneficial in improving nutrient uptake for plant growth and photosynthesis in plant cultivation.^110^ However, studies have also shown that high levels of citric acid lower the pH of the soil and reduce chlorophyll pigment levels. At high levels, citric acid works as a weak chelating agent, which decreases the uptake of essential macronutrients, which are critical for chlorophyll synthesis.^111,112^ These results are in line with the low antioxidant activity of the samples (see below) with 5 wt % CA (Figure 7, first cycle). Low antioxidant efficiency reduces the protection of plants from oxidative stress (extreme temperature, light intensity and drought).^100,101^ Chlorophylls A and B are complex green pigments found in plants, fruits, algae, and certain bacteria.^113,114^ Efficient methods for extracting small amounts of CHL from leaf tissue have been developed to study the photosynthesis process, nutrient effects and environmental stresses in plants.^115−117^ Chlorophyll works by absorbing sun-light and converting it into chemical energy, serving as a primary energy source for plants.^118^ Plants with more pigments are typically better equipped to absorb and utilize sun-light energy efficiently, which contributes to their growth. This increase in pigmentation enhances the plant growth rate efficacy by prolonged germination time (Figure S18). The reference samples showed practically constant levels of pigments within both GR periods (10 and 20 days), which suggests that the roots of the plants under these conditions experience a limitation in their growth as a consequence of a soil deficient in nutrients and biological activity, as well as, high abiotic stress.^119^

### 3.5 Antioxidant and Antibacterial Properties

All WG-based materials showed antioxidant activity to a certain extent (Figure 7). The overall highest antioxidant activity was observed for WG/G/ABC/1GA and WG/G/ABC/5GA. Gallic acid is well-known for its radical scavenging properties.^120^ Both foams showed a radical scavenging efficiency close to that observed for pure gallic acid, especially after the third cycle. In the case of citric acid, the sample with 5 wt % of it (WG/G/ABC/5CA) showed a lower antioxidant effect (first cycle) than that with 1 wt % (WG/G/ABC/1CA), indicating that high amounts of citric acid have adverse effects.

The use of GNP also increased the antioxidant activity, which is consistent with bioassimilation results. A similar finding has also been reported for chitosan/GNP films.^107,121^ In comparison to the antioxidant activity of WG, GNP showed poor antioxidant activity by itself (Table S4). However, a synergetic behavior between WG and GNP is evident. Cross-linking of WG with GNP might favor the exposure of charged amino acid residues. In addition, it may lead to the formation of carboxyl groups from hydrolysis of the methyl ester group of GNP.^18^ These groups can participate actively in redox reactions. Another interesting point is that the cross-linking involving GNP reduces its possible release into the solution, which is observed along the oxidative cycles, preventing the oxidative process within the material and its degradation.^107,122^ This result is beneficial for material applications that require high oxidative resistance at the material surface.

The antibacterial properties of the WG foams against *E. coli* and *B. cereus* after 24 h of incubation is shown in Figure S19. Bacterial growth was not observed on the material’s surface in the case of both Gram-negative (*E. coli*) and Gram-positive (*B. cereus*) pathogen, even for WG with only glycerol added, indicating that the short-term antibacterial activity of the WG-based porous materials is mainly associated with the polymeric matrix. This result may be related to the presence of charged amino acid residues in WG.^123^ However, for the samples with high content of gallic acid (WG/G/ABC/5GA), and those with genipin (WG/G/ABC/1GNP and WG/G/ABC/5GNP), bacterial growth of Gram-negative (*E. coli*) was observed (Figure S19I.d,g,h).

### 3.6 Recycling Properties

**Figure 8:**
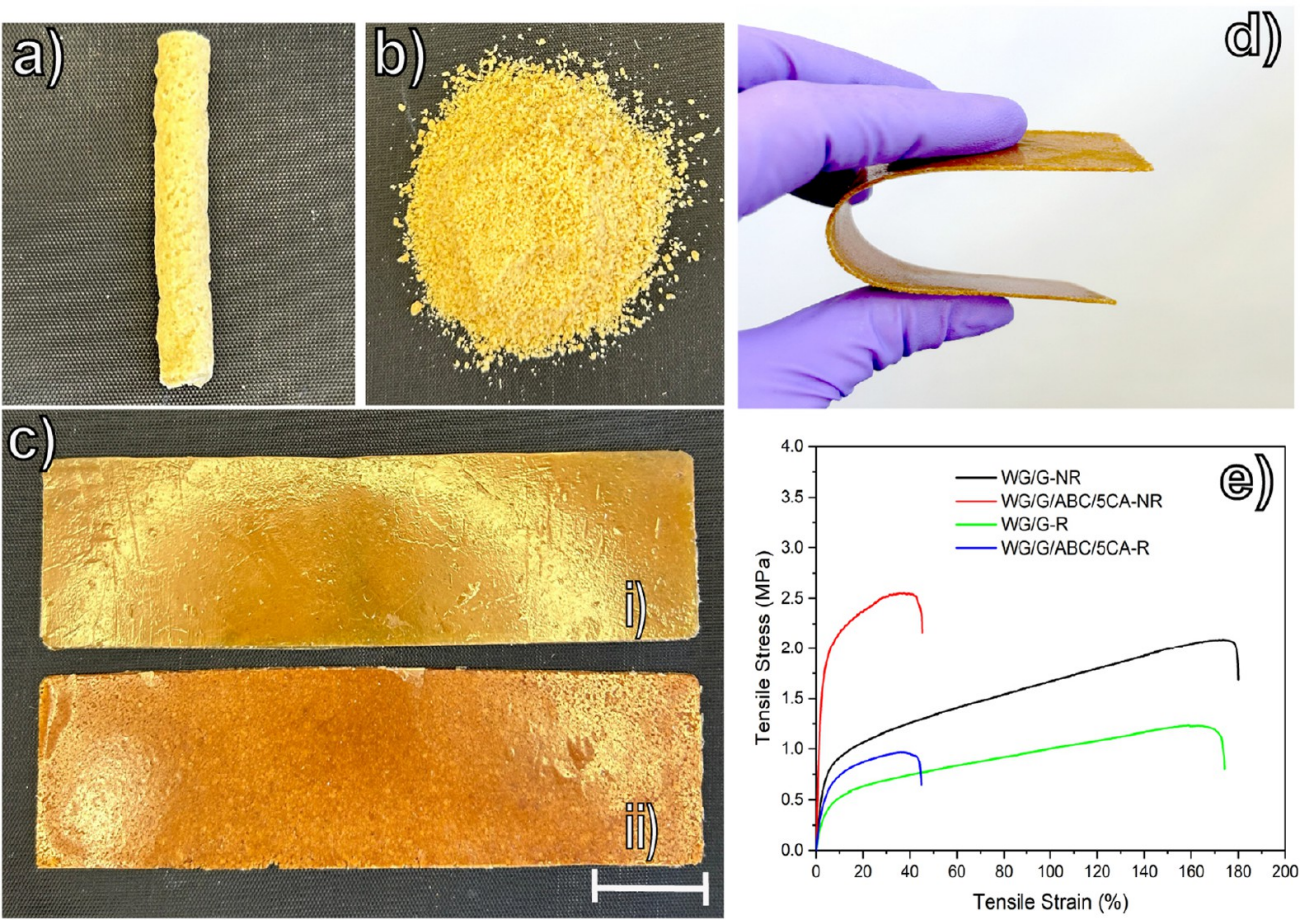
WG/G/ABC/5CA (a) foam and (b) ground material. (c) Compression-molded recycled films: (i) WG/G-R, and (ii) WG/G/ABC/5CA-R. (d) Image illustrating the high flexibility of the WG/G/ABC/5CA recycled film, and (e) representative tensile curves of recycled and non-recycled samples. Note: The scale bar in (c) is 3 cm.

Figure 8 shows the ability of the WG foams (exemplified with WG/G and WG/G/ABC/5CA (the foam with the lowest density^123^) to be recycled into new products. The foams were first ground and then compression molded into films, and the non-recycled material was compression molded directly into films (Figure 8a–c). All four films showed good flexibility, exemplified in Figure 8d. The non-recycled WG/G material was significantly stronger (yield and fracture strength) and tougher than the recycled WG/G (Figure 8e). The stiffness (modulus) and ductility (elongation at break) were also higher for the former sample, although the differences were not significantly different (Table 2). For the samples with citric acid, the non-recycled material was stiffer and stronger than the recycled material, but the toughness and ductility were not significantly different. In fact, the recycled citric-acid sample tended to have a larger elongation at break than the non-recycled sample (Figure 8e). The differences in mechanical properties of the recycled and non-recycled samples are probably due to several factors. One factor is changes in the cross-link network after a thermal treatment. In contrast to common synthetic thermoplastics, protein plastics contain disulfide cross-links, and often dityrosine cross-links, and these may be broken and reformed in other molecular geometries under thermal treatment. The lowering in strength suggests that the cross-link density decreased in the recycling process. Isopeptide bonds may develop sparked by successive thermal treatments.^124^ These would, however, result in stronger recycled materials, which was not observed. Notably, the sample with citric acid (WG/G/ABC/5CA) was overall stiffer and stronger at yield but more brittle and less ductile than the citric-acid-free sample (WG/G) in both nonrecycled and recycled conditions. This is ascribed to citric acid specific cross-linking.^21^ It should also be mentioned that the reaction products from the dissociation of ABC did not seem to have any affects on the compression molded (non-recycled) film since no porosity was observed (Figure S20a.I,a.II,b.I,b.II). Hence the reaction products of ABC were outgassed during the pressing cycle without expanding the material (Figure S20b). The results show that WG foams can be recycled into new products as an alternative to direct biodegradation. However, care should be taken when deciding on new products. Downcycling into less demanding products, for instance, various types of covers and plugs, is probably the most realistic scenario.

**Table 2:** Mechanical Properties of the Films^a^

samples	*E* (MPa)	σ_y_ (MPa)	*U* (MJ m^–3^)	σ_b_ (MPa)	ε_b_ (%)
WG/G-NR	24 ± 10 ^ab^	0.42 ± 0.16 ^b^	3.15 ± 0.42 ^b^	2.20 ± 0.34 ^b^	185 ± 30 ^b^
WG/G/ABC/5CA-NR	89 ± 43^c^	1.52 ± 0.73 ^c^	0.57 ± 0.27 ^a^	2.47 ± 0.80 ^b^	29 ± 14 ^a^
WG/G-R	11 ± 3 ^a^	0.19 ± 0.06 ^a^	0.92 ± 0.43 ^a^	1.05 ± 0.40 ^a^	114 ± 59 ^b^
WG/G/ABC/5CA-R	21 ± 1 ^b^	0.36 ± 0.02 ^b^	0.48 ± 0.18 ^a^	1.05 ± 0.09 ^a^	54 ± 15 ^a^

a The nomenclature refers to *E* (Young’s modulus), σ_y_ (yield stress at 10% strain), *U* (Toughness), σ_b_ (stress at break), ε_b_ (elongation at break). Note: Different letters mean that one column’s values are significantly different (*P* < 0.05).

### 3.7 Climate Impact Mitigation

On a weight basis, the global warming potential (GWP) of the plasticized WG-based foams was generally 60–70% lower compared to the reference NBR material (Figure S21). While fossil carbon content in the reference material contributed to 43% of the total emissions, end-of-life fossil carbon release in the WG-based foams only amounted to between 0 and 1.4%. The main sources of emissions contributing to the GWP of the WG-based foams were the production of wheat gluten and glycerol. However, it is important to mention that no GPW data was available for genipin, which would increase the GWP of the foams containing genipin. GHG emission savings are usually achieved by e.g., using low GWP materials and replacing materials with higher GWP, on a mass basis. In the present case, WG-based foams had a much higher density: 640–950 kg/m^3^ compared to the reference NBR (120 kg/m^3^).^21^ This leads to a strong increase in the amount of material required to replace materials on a per-volume basis, which makes it currently unfavorable from an environmental and even an economic point of view. Future work should, therefore, focus on producing lower-density WG foams.

From a life-cycle perspective, the impact of the tested materials is also dependent on to what degree materials can be reused or recycled. For conventional neat NBR, current research efforts focus on recycling residual virgin materials that occur in product manufacturing.^125^ However, it is currently difficult to reuse vulcanized rubbers, and incineration is a common end-of-life scenario for these. To our knowledge, the reuse and recycling of WG-based foams have not been investigated. However, since such foam-based products may end up in the environment or landfills, biodegradability may currently play a more important role. In fact, these wheat gluten-based materials are the focus of mold resistance and soil biodegradation studies, which show that product shelf life increases with a higher process temperature and pressure and with the use of certain foaming agents, such as ABC.^28^ On the other hand, WG-based foams have been shown to be readily biodegradable in suitable environments and degrade faster than common biobased and biodegradable materials like polylactic acid (PLA).^7^ The results of the biodegradation tests performed in the present study support such a claim.

### 3.8 Industrial Relevance and Scalability of the Foams

Beyond their biodegradability and bioassimilation, WG foams also possess potential industrial and commercial uses with their scalable production and sustainability benefits. The foams were manufactured through extrusion, a common polymer processing method, which showed that they may be produced in large quantities and high rates.^7−11,126^ In contrast to most biodegradable plastics, which only decompose under special conditions,^127^ WG foams decompose effectively in soil and aqueous environments and are therefore suitable for single-use items like biodegradable single-use sanitary pads, in packagings, and agricultural mulch films (Figures 2, 5, S1 and S8). One of the benefits of WG foams is that they provide several sustainability advantages: not only do they readily degrade, but they also enhance soil quality through the introduction of organic nitrogen into the environment, as demonstrated by the plant growth pattern of coriander in this study (Figures S14–S18). It is this bioassimilation characteristic that positions WG foams as viable substitutes for synthetic agricultural films and soil conditioners, particularly for horticulture and controlled agricultural systems. In addition, WG foams have the ability to be recycled using thermoplastic processing techniques before they are finally considered for the end-of-life composting/biodegradation (Figure 8). However, to expand the possible applications, future efforts should focus on reducing the density of the foams and improve the moisture resistance (especially for warm and humid (tropical) environments).

## Data Availability

Data will be made available on request.

## Supporting Information Available

Development of mold in or ontop of the soil; full FTIR spectra of the NBR foam in different environments; SEM cross sections of extruded samples after 7 weeks in soil; additional results on hydrolysis in acidic, neutral, and alkaline conditions including weight loss, pH development and electropherogram data; full FTIR and amide I spectra under hydrolytic degradation; cross sections of extruded samples revealed by SEM before and after hydrolytic degradation; illustration of microbial activity during high humidity environment; development, indicators of vegetal growth and illustrations of vegetative growth; UV spectra of coriander plant after germination; content of pigment in the coriander leaves during germination; radical scavenging activity of WG and additives used and images showing antibacterial activity against (I) *E. coli* and (II) *B. cereus*; microstructure and FTIR spectra of recycled materials; climat impact mitigation and final stage carbon release (PDF)
The Supporting Information includes components used in a foamed nitrile butadiene rubber, global warming potential (GWP); results on biodegradation in soil, illustration of microbial activity during soil degradation (Video S1) (MP4)

## References

[ref1] Ashby M. F.; Gibson L. J. Cellular Solids: Structure and Properties, 2nd ed.; Cambridge University Press: Cambridge, 1997.

[ref2] Goel V.; Luthra P.; Kapur G. s.; Ramakumar S. Biodegradable/Bio-plastics: Myths and Realities. J. Polym. Environ. 2021, 29, 3079–3104. 10.1007/s10924-021-02099-1.

[ref3] Halada K. Progress of ecomaterials toward a sustainable society. Curr. Opin. Solid State Mater. Sci. 2003, 7, 209–216. 10.1016/j.cossms.2003.09.007.

[ref4] Jayasekara R.; Sheridan S.; Lourbakos E.; Beh H.; Christie G. B. Y.; Jenkins M.; Halley P. B.; McGlashan S.; Lonergan G. T. Biodegradation and ecotoxicity evaluation of a bionolle and starch blend and its degradation products in compost. Int. Biodeterior. Biodegrad. 2003, 51 (1), 77–81. 10.1016/S0964-8305(02)00090-2.

[ref5] Blomfeldt T. O. J.; Olsson R. T.; Menon M.; Plackett D.; Johansson E.; Hedenqvist M. S. Novel Foams Based on Freeze-Dried Renewable Vital Wheat Gluten. Macromol. Mater. Eng. 2010, 295 (9), 796–801. 10.1002/mame.201000049.

[ref6] Mauricio-Iglesias M.; Peyron S.; Guillard V.; Gontard N. Wheat gluten nanocomposite films as food-contact materials: Migration tests and impact of a novel food stabilization technology (high pressure). J. Appl. Polym. Sci. 2010, 116 (5), 2526–2535. 10.1002/app.31647.

[ref7] Bettelli M. A.; Capezza A. J.; Nilsson F.; Johansson E.; Olsson R. T.; Hedenqvist M. S. Sustainable Wheat Protein Biofoams: Dry Upscalable Extrusion at Low Temperature. Biomacromolecules 2022, 23, 5116–5126. 10.1021/acs.biomac.2c00953. 36349363 PMC9748940

[ref8] Capezza A. J.; Robert E.; Lundman M.; Newson W. R.; Johansson E.; Hedenqvist M. S.; Olsson R. T. Extrusion of Porous Protein-Based Polymers and Their Liquid Absorption Characteristics. Polymers 2020, 12 (2), 459–517. 10.3390/polym12020459. 32079125 PMC7077648

[ref9] Cho S. W.; Gällstedt M.; Johansson E.; Hedenqvist M. S. Injection-molded nanocomposites and materials based on wheat gluten. Int. J. Biol. Macromol. 2011, 48 (1), 146–152. 10.1016/j.ijbiomac.2010.10.012. 21035504

[ref10] Gällstedt M.; Mattozzi A.; Johansson E.; Hedenqvist M. S. Transport and Tensile Properties of Compression-Molded Wheat Gluten Films. Biomacromolecules 2004, 5 (5), 2020–2028. 10.1021/bm040044q. 15360319

[ref11] Ullsten N. H.; Cho S.-W.; Spencer G.; Gällstedt M.; Johansson E.; Hedenqvist M. S. Properties of Extruded Vital Wheat Gluten Sheets with Sodium Hydroxide and Salicylic Acid. Biomacromolecules 2009, 10 (3), 479–488. 10.1021/bm800691h. 19178277

[ref12] Davis G.; Read A.; Bulson H.; Harrison D.; Billett E. Open windrow composting of polymers: an investigation into the rate of degradation of polyethylene. Resour., Conserv. Recycl. 2004, 40 (4), 343–357. 10.1016/S0921-3449(03)00086-7.

[ref13] Wu Q.; Andersson R. L.; Holgate T.; Johansson E.; Gedde U. W.; Olsson R. T.; Hedenqvist M. S. Highly porous flame-retardant and sustainable biofoams based on wheat gluten and in situ polymerized silica. J. Mater. Chem. A 2014, 2 (48), 20996–21009. 10.1039/C4TA04787G.

[ref14] Wu Q.; Sundborg H.; Andersson R. L.; Peuvot K.; Guex L.; Nilsson F.; Hedenqvist M. S.; Olsson R. T. Conductive biofoams of wheat gluten containing carbon nanotubes, carbon black or reduced graphene oxide. RSC Adv. 2017, 7 (30), 18260–18269. 10.1039/C7RA01082F.

[ref15] Capezza A. J.; Glad D.; Özeren H. D.; Newson W. R.; Olsson R. T.; Johansson E.; Hedenqvist M. S. Novel Sustainable Superabsorbents: A One-Pot Method for Functionalization of Side-Stream Potato Proteins. ACS Sustain. Chem. Eng. 2019, 7 (21), 17845–17854. 10.1021/acssuschemeng.9b04352.

[ref16] Capezza A. J.; Lundman M.; Olsson R. T.; Newson W. R.; Hedenqvist M. S.; Johansson E. Carboxylated Wheat Gluten Proteins: A Green Solution for Production of Sustainable Superabsorbent Materials. Biomacromolecules 2020, 21 (5), 1709–1719. 10.1021/acs.biomac.9b01646. 31899621

[ref17] Capezza A. J.; Newson W. R.; Olsson R. T.; Hedenqvist M. S.; Johansson E. Advances in the Use of Protein-Based Materials: Toward Sustainable Naturally Sourced Absorbent Materials. ACS Sustain. Chem. Eng. 2019, 7 (5), 4532–4547. 10.1021/acssuschemeng.8b05400.

[ref18] Capezza A. J.; Wu Q.; Newson W. R.; Olsson R. T.; Espuche E.; Johansson E.; Hedenqvist M. S. Superabsorbent and Fully Biobased Protein Foams with a Natural Cross-Linker and Cellulose Nanofibers. ACS Omega 2019, 4 (19), 18257–18267. 10.1021/acsomega.9b02271. 31720526 PMC6844118

[ref19] Das O.; Rasheed F.; Kim N. K.; Johansson E.; Capezza A. J.; Kalamkarov A. L.; Hedenqvist M. S. The development of fire and microbe resistant sustainable gluten plastics. J. Clean. Prod. 2019, 222, 163–173. 10.1016/j.jclepro.2019.03.032.

[ref20] Wu Q.; Yu S.; Kollert M.; Mtimet M.; Roth S. V.; Gedde U. W.; Johansson E.; Olsson R. T.; Hedenqvist M. S. Highly Absorbing Antimicrobial Biofoams Based on Wheat Gluten and Its Biohybrids. ACS Sustain. Chem. Eng. 2016, 4 (4), 2395–2404. 10.1021/acssuschemeng.6b00099.

[ref21] Bettelli M. A.; Hu Q.; Capezza A. J.; Johansson E.; Olsson R. T.; Hedenqvist M. S. Effects of multi-functional additives during foam extrusion of wheat gluten materials. Commun. Chem. 2024, 7 (1), 75 10.1038/s42004-024-01150-1. 38570707 PMC10991538

[ref22] Reubsaet J. L.; Beijnen J. H.; Bult A.; van Maanen R. J.; Marchal J. A.; Underberg W. J. Analytical techniques used to study the degradation of proteins and peptides: physical instability. J. Pharm. Biomed. Anal. 1998, 17 (6–7), 979 10.1016/S0731-7085(98)00064-8. 9884188

[ref23] Whitaker J. R.; Feeney R. E.; Sternberg M. M. Chemical and physical modification of proteins by the hydroxide ion. Crit. Rev. Food Sci. Nutr. 1983, 19 (3), 173–212. 10.1080/10408398309527375. 6380954

[ref24] Domenek S.; Feuilloley P.; Gratraud J.; Morel M.-H.; Guilbert S. Biodegradability of wheat gluten based bioplastics. Chemosphere 2004, 54 (4), 551–559. 10.1016/S0045-6535(03)00760-4. 14581057

[ref25] Park S. K.; Hettiarachchy N. S.; Were L. Degradation Behavior of Soy Protein–Wheat Gluten Films in Simulated Soil Conditions. J. Agric. Food Chem. 2000, 48 (7), 3027–3031. 10.1021/jf0000272. 10898660

[ref26] John J.; Tang J.; Bhattacharya M. Processing of biodegradable blends of wheat gluten and modified polycaprolactone. Polymer 1998, 39 (13), 2883–2895. 10.1016/S0032-3861(97)00553-3.

[ref27] Zhang X.; Gozukara Y.; Sangwan P.; Gao D.; Bateman S. Biodegradation of chemically modified wheat gluten-based natural polymer materials. Polym. Degrad. Stab. 2010, 95 (12), 2309–2317. 10.1016/j.polymdegradstab.2010.09.001.

[ref28] Bettelli M. A.; Traissac E.; Latras A.; Rosado M. J.; Guerrero A.; Olsson R. T.; Hedenqvist M. S.; Capezza A. J. Eco-friendly disposable porous absorbents from gluten proteins through diverse plastic processing techniques. J. Clean. Prod. 2024, 459, 142419–142516. 10.1016/j.jclepro.2024.142419.

[ref29] Blomfeldt T. O. J.; Kuktaite R.; Johansson E.; Hedenqvist M. S. Mechanical Properties and Network Structure of Wheat Gluten Foams. Biomacromolecules 2011, 12 (5), 1707–1715. 10.1021/bm200067f. 21413807

[ref30] Türe H.; Gällstedt M.; Kuktaite R.; Johansson E.; Hedenqvist M. S. Protein network structure and properties of wheat gluten extrudates using a novel solvent-free approach with urea as a combined denaturant and plasticiser. Soft Matter 2011, 7 (19), 9416–9423. 10.1039/c1sm05830d.

[ref31] Vetter J. L. Leavening Agents. In Encyclopedia of Food Sciences and Nutrition; Caballero B., Ed.; Academic Press: Oxford, 2003.

[ref32] Capezza A. J.; Bettelli M.; Wei X.; Jiménez-Rosado M.; Guerrero A.; Hedenqvist M. Biodegradable Fiber-Reinforced Gluten Biocomposites for Replacement of Fossil-Based Plastics. ACS Omega 2024, 9 (1), 1341–1351. 10.1021/acsomega.3c07711. 38222641 PMC10785611

[ref33] Jiménez-Rosado M.; Perez-Puyana V.; Sánchez-Cid P.; Guerrero A.; Romero A. Incorporation of ZnO nanoparticles into soy protein-based bioplastics to improve their functional properties. Polymers 2021, 13 (4), 486 10.3390/polym13040486. 33557059 PMC7913798

[ref34] Notizie A. Nitrophoska Special 12 + 12 + 17. Da sempre e per sempre. https://agronotizie.imagelinenetwork.com/fertilizzazione/2015/10/08/nitrophoskasupregsup-special-121217-da-sempre-e-per-sempre/45737 (accessed June 08, 2024).

[ref35] Market, G. s. Biohumus Max-Humvit—100% organiskt vermikompostgödselmedel—Target—5 liter. https://gardenseedsmarket.com/biohumus-max-humvit-100-organisktvermikompostgoedselmedel-target-5-liter.html?currency=SEK&gad_source=1&gclid=CjwKCAiAxea5BhBeEiwAh4t5K9eZMB9b0TKgCRXsPBRHuwftuqvtnQZKT4RhLg1KsCnyP_WDruyDxBoCs4YQAvD_BwE# (accessed June 08, 2024).

[ref36] Silva R. R.; Marques C. S.; Arruda T. R.; Teixeira S. C.; de Oliveira T. V. Biodegradation of Polymers: Stages, Measurement, Standards and Prospects. Macromol. 2023, 3 (2), 371–399. 10.3390/macromol3020023.

[ref37] Guo L.; Wang Y.; Wang M.; Shaghaleh H.; Hamoud Y. A.; Xu X.; Liu H. Synthesis of bio-based MIL-100(Fe)@CNF-SA composite hydrogel and its application in slow-release N-fertilizer. J. Clean. Prod. 2021, 324, 129274 10.1016/j.jclepro.2021.129274.

[ref38] Agrotendencia Agroshow La expo del agro online. https://agroshow.info/(accessed May 30, 2024).

[ref39] Briassoulis D.; Dejean C. Critical review of norms and standards for biodegradable agricultural plastics part Ι. Biodegradation in soil. J. Polym. Environ. 2010, 18, 384–400. 10.1007/s10924-010-0168-1.

[ref40] Sofo A.; Elshafie H.; Camele I. Structural and Functional Organization of the Root System: A Comparative Study on Five Plant Species. Plants 2020, 9, 1338 10.3390/plants9101338. 33050531 PMC7601878

[ref41] Mackinney G. Absorption of Light by Chlorophyll Solutions. J. Biol. Chem. 1941, 140 (2), 315–322. 10.1016/S0021-9258(18)51320-X.

[ref42] Kirk J. T.; Allen R. L. Dependence of chloroplast pigment synthesis on protein synthesis: effect of actidione. Biochem. Biophys. Res. Commun. 1965, 21 (6), 523 10.1016/0006-291x(65)90516-4. 5879460

[ref43] Brand-Williams W.; Cuvelier M.-E.; Berset C. Use of a free radical method to evaluate antioxidant activity. Lebensm. Wiss. Technol. 1995, 28 (1), 25–30. 10.1016/S0023-6438(95)80008-5.

[ref44] Keke G.; Hao L.; Xiaoyang Z.; Ke H.; Li G.; Yan G.; Beibei Q.; Feifei Z.; Jian H. Preparation method of nitrile butadiene rubber yoga mat, 2013.

[ref45] Jugé A.; Moreno-Villafranca J.; Perez-Puyana V. M.; Jiménez-Rosado M.; Sabino M.; Capezza A. J. Porous Thermoformed Protein Bioblends as Degradable Absorbent Alternatives in Sanitary Materials. ACS Appl. Polym. Mater. 2023, 5, 6976–6989. 10.1021/acsapm.3c01027. 37705711 PMC10497054

[ref46] Wernke M. J. Glycerol. In Encyclopedia of Toxicology; Academic Press, 2014.

[ref47] Rineau F.; Stas J.; Nguyen N. H.; Kuyper T. W.; Carleer R.; Vangronsveld J.; Colpaert J. V.; Kennedy P. G. Ectomycorrhizal Fungal Protein Degradation Ability Predicted by Soil Organic Nitrogen Availability. Appl. Environ. Microbiol. 2016, 82 (5), 1391–1400. 10.1128/AEM.03191-15. PMC4771325 26682855

[ref48] California S. o., CalRecycle. Obtenido de Compost Pile Microbes. 2022.

[ref49] Ling M.; Qi M.; Li S.; Shi Y.; Pan Q.; Cheng C.; Yang W.; Duan C. The influence of polyphenol supplementation on ester formation during red wine alcoholic fermentation. Food Chem. 2022, 377 (131961), 131961 10.1016/j.foodchem.2021.131961. 34990947

[ref50] Mauricio J. C.; Moreno J. J.; Valero E. M.; Zea L.; Medina M.; Ortega J. M. Ester formation and specific activities of in vitro alcohol acetyltransferase and esterase by Saccharomyces cerevisiae during grape must fermentation. J. Agric. Food Chem. 1993, 41 (11), 2086–2091. 10.1021/jf00035a050.

[ref51] Wieser H.; Gutser R.; von Tucher S. Influence of sulphur fertilisation on quantities and proportions of gluten protein types in wheat flour. J. Cereal. Sci. 2004, 40 (3), 239–244. 10.1016/j.jcs.2004.05.005.

[ref52] Zhang L.; Qiu Y.-Y.; Sharma K. R.; Shi T.; Song Y.; Sun J.; Liang Z.; Yuan Z.; Jiang F. Hydrogen sulfide control in sewer systems: A critical review of recent progress. Water Res. 2023, 240 (120046), 120046 10.1016/j.watres.2023.120046. 37224665

[ref53] Karpe A. V.; Beale D. J.; Godhani N. B.; Morrison P. D.; Harding I. H.; Palombo E. A. Untargeted Metabolic Profiling of Winery-Derived Biomass Waste Degradation by Penicillium chrysogenum. J. Agric. Food Chem. 2015, 63 (49), 10696 10.1021/acs.jafc.5b04834. 26611372

[ref54] Sobolev V.; Arias R.; Goodman K.; Walk T.; Orner V.; Faustinelli P.; Massa A. Suppression of Aflatoxin Production in Aspergillus Species by Selected Peanut (Arachis hypogaea) Stilbenoids. J. Agric. Food Chem. 2018, 66 (1), 118–126. 10.1021/acs.jafc.7b04542. 29207242

[ref55] Tang J. L.; Zhou Z. Y.; Yang T.; Yao C.; Wu L. W.; Li G. Y. Azaphilone Alkaloids with Anti-inflammatory Activity from Fungus Penicillium sclerotiorum cib-411. J. Agric. Food Chem. 2019, 67 (8), 2175–2182. 10.1021/acs.jafc.8b05628. 30702881

[ref56] Tong J.; Wu H.; Jiang X.; Ruan C.; Li W.; Zhang H.; Pan S.; Wang J.; Ren J.; Zhang C.; Shi J. Dual Regulatory Role of Penicillium oxalicum SL2 in Soil: Phosphorus Solubilization and Pb Stabilization. Environ. Sci. Technol. 2024, 58 (1), 603–616. 10.1021/acs.est.3c08881. 38109294

[ref57] Kumar Sen S.; Raut S. Microbial degradation of low density polyethylene (LDPE): A review. J. Environ. Chem. Eng. 2015, 3 (1), 462–473. 10.1016/j.jece.2015.01.003.

[ref58] Montazer Z.; Najafi M. B. H.; Levin D. B. Challenges with Verifying Microbial Degradation of Polyethylene. Polymers 2020, 12 (1), 123–124. 10.3390/polym12010123. 31948075 PMC7022683

[ref59] Cori C. F. Metabolic Pathways in Microorganisms. J. Am. Chem. Soc. 1962, 84 (8), 1518 10.1021/ja00867a062.

[ref60] Sonenshein A. The Krebs Citric Acid Cycle. In Bacillus subtilis and its Closest Relatives: from Genes to Cells; Wiley Online Library, 2014; pp 151–162.

[ref61] Parveen S.; Chaudhury P.; Dasmahapatra U.; Dasgupta S. Biodegradable protein films from gallic acid and the cataractous eye protein isolate. Int. J. Biol. Macromol. 2019, 139, 12–20. 10.1016/j.ijbiomac.2019.07.143. 31369779

[ref62] Pinanjota J.; Rodríguez A.; Santacruz C. Energy conversion efficiency of genipin-based dye sensitized solar cells. AIP Conf. Proc. 2018, 2003, 020012 10.1063/1.5050364.

[ref63] Deng L.; Li Y.; Feng F.; Zhang H. Study on wettability, mechanical property and biocompatibility of electrospun gelatin/zein nanofibers cross-linked by glucose. Food Hydrocolloids 2019, 87, 1–10. 10.1016/j.foodhyd.2018.07.042.

[ref64] Azevedo V. M.; Borges S. V.; Marconcini J. M.; Yoshida M. I.; Neto A. R. S.; Pereira T. C.; Pereira C. F. G. Effect of replacement of corn starch by whey protein isolate in biodegradable film blends obtained by extrusion. Carbohydr. Polym. 2017, 157, 971–980. 10.1016/j.carbpol.2016.10.046. 27988016

[ref65] Ávila-Martín L.; Beltrán-Osuna A. ´. A.; Perilla J. E. Effect of the Addition of Citric Acid and Whey Protein Isolate in Canna indica L. Starch Films Obtained by Solvent Casting. J. Polym. Environ. 2020, 28 (3), 871–883. 10.1007/s10924-019-01648-z.

[ref66] Dixit V.; Tewari J. C.; Cho B.-K.; Irudayaraj J. M. K. Identification and Quantification of Industrial Grade Glycerol Adulteration in Red Wine with Fourier Transform Infrared Spectroscopy Using Chemometrics and Artificial Neural Networks. Appl. Spectrosc. 2005, 59 (12), 1553–1561. 10.1366/000370205775142638. 16390596

[ref67] Gedde U. W.; Hedenqvist M. S. Fundamental Polymer Science; Springer, 2019.

[ref68] Vasylieva A.; Doroshenko I.; Vaskivskyi Y.; Chernolevska Y.; Pogorelov V. FTIR study of condensed water structure. J. Mol. Struct. 2018, 1167, 232–238. 10.1016/j.molstruc.2018.05.002.

[ref69] Guimarães J.; Cursino A.; Saul C.; Sierakowski M.; Pereira Ramos L.; Satyanarayana K. G. Evaluation of Castor Oil Cake Starch and Recovered Glycerol and Development of “Green” Composites Based on Those with Plant Fibers. Materials 2016, 9 (76), 1–18. 10.3390/ma9020076. PMC5456479 28787878

[ref70] Poh B. T.; Ismail H.; Quah E. H.; Chin P. L. Cure and mechanical properties of filled SMR L/ENR 25 and SMR L/SBR blends. J. Appl. Polym. Sci. 2001, 81 (1), 47–52. 10.1002/app.1411.

[ref71] Zuiderduin W. C. J.; Westzaan C.; Huétink J.; Gaymans R. J. Toughening of polypropylene with calcium carbonate particles. Polymer 2003, 44 (1), 261–275. 10.1016/S0032-3861(02)00769-3.

[ref72] Da Silva A. L. N.; Rocha M. C. G.; Moraes M. A. R.; Valente C. A. R.; Coutinho F. M. B. Mechanical and rheological properties of composites based on polyolefin and mineral additives. Polym. Test. 2002, 21 (1), 57–60. 10.1016/S0142-9418(01)00047-2.

[ref73] Jolanta K.; Nowak B.; Karcz J. Biodegradation of Pre-Aged Modified Polyethylene Films. In Scanning Electron Microscopy; IntechOpent, 2012.

[ref74] Rozali S. N. M.; Milani E. A.; Deed R. C.; Silva F. V. M. Bacteria, mould and yeast spore inactivation studies by scanning electron microscope observations. Int. J. Food Microbiol. 2017, 263, 17–25. 10.1016/j.ijfoodmicro.2017.10.008. 29024903

[ref75] Huang Z.; Hua W.; Verreault D.; Allen H. C. Salty glycerol versus salty water surface organization: bromide and iodide surface propensities. J. Phys. Chem. A 2013, 117 (29), 6346 10.1021/jp4020228. 23663033

[ref76] Velez A. R.; Mufari J. R.; Rovetto L. J. Sodium salts solubility in ternary glycerol+water+alcohol mixtures present in purification process of crude glycerol from the biodiesel industry. Fluid Phase Equilib. 2019, 497, 55–63. 10.1016/j.fluid.2019.05.023.

[ref77] Arancibia M. Y.; López-Caballero M. E.; Gómez-Guillén M. C.; Montero P. Release of volatile compounds and biodegradability of active soy protein lignin blend films with added citronella essential oil. Food Control 2014, 44, 7–15. 10.1016/j.foodcont.2014.03.025.

[ref78] Majzoobi M. Effects of pH changes on functional properties of native and acetylated wheat gluten. Int. Food Res. J. 2013, 21 (3), 1219–1224.

[ref79] Selle P. H.; Cowieson A. J.; Cowieson N. P.; Ravindran V. Protein-phytate interactions in pig and poultry nutrition: a reappraisal. Nutr. Res. Rev. 2012, 25 (1), 1–17. 10.1017/S0954422411000151. 22309781

[ref80] Zhang A.-Q.; Li X.-Y.; Han Y.-N.; Liu B.-H.; Zhang H.-L.; Gao J.-H.; Zhang Y.-H. Improving interface properties of zein hydrolysis and its application in salad dressing through dispersion improvement assisted by potassium oleate aqueous solution. Food Hydrocolloids 2022, 130 (107719), 107719 10.1016/j.foodhyd.2022.107719.

[ref81] Akhlaghi S.; Hedenqvist M. S.; Braña M. T. C.; Bellander M.; Gedde U. W. Deterioration of acrylonitrile butadiene rubber in rapeseed biodiesel. Polym. Degrad. Stab. 2015, 111, 211–222. 10.1016/j.polymdegradstab.2014.11.012.

[ref82] Miles C. E.; Lima M. R. N.; Buevich F.; Gwin C.; Sanjeeva Murthy N.; Kohn J. Comprehensive hydrolytic degradation study of a new poly(ester-amide) used for total meniscus replacement. Polym. Degrad. Stab. 2021, 190 (109617), 109617 10.1016/j.polymdegradstab.2021.109617.

[ref83] Hofmann D.; Entrialgo-Castaño M.; Kratz K.; Lendlein A. Knowledge-based approach towards hydrolytic degradation of polymer-based biomaterials. Adv. mater. 2009, 21 (32–33), 3237–3245. 10.1002/adma.200802213. 20882494

[ref84] Pischedda A.; Tosin M.; Degli-Innocenti F. Biodegradation of plastics in soil: The effect of temperature. Polym. Degrad. Stab. 2019, 170 (109017), 109017 10.1016/j.polymdegradstab.2019.109017.

[ref85] Briassoulis D.; Mistriotis A. Key parameters in testing biodegradation of bio-based materials in soil. Chemosphere 2018, 207, 18–26. 10.1016/j.chemosphere.2018.05.024. 29763763

[ref86] Kabir E.; Kaur R.; Lee J.; Kim K.-H.; Kwon E. E. Prospects of biopolymer technology as an alternative option for non-degradable plastics and sustainable management of plastic wastes. J. Clean. Prod. 2020, 258 (120536), 120536 10.1016/j.jclepro.2020.120536.

[ref87] Cabra V.; Arreguin R.; Vazquez-Duhalt R.; Farres A. Effect of alkaline deamidation on the structure, surface hydrophobicity, and emulsifying properties of the Z19 α-zein. J. Agric. Food Chem. 2007, 55 (2), 439–445. 10.1021/jf061002r. 17227077

[ref88] Campobenedetto C.; Mannino G.; Beekwilder J.; Contartese V.; Karlova R.; Bertea C. M. The application of a biostimulant based on tannins affects root architecture and improves tolerance to salinity in tomato plants. Sci. Rep. 2021, 11 (1), 354–415. 10.1038/s41598-020-79770-5. 33432010 PMC7801735

[ref89] Negi A. S.; Darokar M. P.; Chattopadhyay S. K.; Garg A.; Bhattacharya A. K.; Srivastava V.; Khanuja S. P. S. Synthesis of a novel plant growth promoter from gallic acid. Bioorg. Med. Chem. Lett. 2005, 15 (4), 1243–1247. 10.1016/j.bmcl.2004.11.079. 15686951

[ref90] Ammineni S. P.; Lingaraju D.; Nagaraju C. Aging characterization and degradation of Nitrile Butadiene Rubber for viscoelastic damping applications. Mater. Today: Proc. 2023, 1–9. 10.1016/j.matpr.2023.08.242.

[ref91] Özeren H. D. Plasticization of Biobased Polymers: A Combined Experimental and Simulation Approach. Ph.D. Thesis, KTH Royal Institute of Technology, Stockholm, Sweden, 2021.

[ref92] Qiu Y.; Zhou Y.; Chang Y.; Liang X.; Zhang H.; Lin X.; Qing K.; Zhou X.; Luo Z. The Effects of Ventilation, Humidity, and Temperature on Bacterial Growth and Bacterial Genera Distribution. Int. Res. J. Publ. Environ. Health 2022, 19 (22), 15345 10.3390/ijerph192215345. PMC9691097 36430064

[ref93] Melgarejo L. M. Experimentos en Fisiología Vegetal, Primera Edición ed.; Universidad Nacional de Colombia, 2010.

[ref94] Fernández F.; Gepts P.; López M. Etapas de Desarrollo en la Planta de Frijol; Investigación y Producción: Frijol, 1985; pp 61–78.

[ref95] Tapia-Hernández J. A.; Madera-Santana T. J.; Rodríguez-Félix F.; Barreras-Urbina C. G. Controlled and Prolonged Release Systems of Urea from Micro- and Nanomaterials as an Alternative for Developing a Sustainable Agriculture: A Review. J. Nanomater. 2022, 5697803, 1–14. 10.1155/2022/5697803.

[ref96] Piedra A. L.; Cepero M. C. G. Indicadores del Crecimiento Inicial y del Estado Nutricional para la Selección Temprana de Genotipos de Arroz (Oryza sativa L.) Tolerantes a la Salinidad. Cultiv. Trop. 2015, 36 (2), 41–48.

[ref97] Dion P.-P.; Jämtgård S.; Bertrand A.; Pepin S.; Dorais M. Organic Nitrogen Uptake and Assimilation in Cucumis sativus Using Position-Specific Labeling and Compound-Specific Isotope Analysis. Front. Plant Sci. 2018, 9, 1–12. 10.3389/fpls.2018.01596. 30459787 PMC6232311

[ref98] Fertilizers N. S. High Nitrogen Fertilizers. https://www.naturesafe.com/knowlegde-center/blog/high-nitrogen-fertilizers (accessed July 02, 2024).

[ref99] Babaei M.; Shabani L.; Hashemi-Shahraki S. Improving the effects of salt stress by β-carotene and gallic acid using increasing antioxidant activity and regulating ion uptake in Lepidium sativum L. Bot. Stud. 2022, 63 (1), 22 10.1186/s40529-022-00352-x. 35840725 PMC9287502

[ref100] Li T.; Hu J.; Tian R.; Wang K.; Li J.; Qayum A.; Bilawal A.; Gantumur M.-A.; Jiang Z.; Hou J. Citric acid promotes disulfide bond formation of whey protein isolate in non-acidic aqueous system. Food Chem. 2021, 338, 127819 10.1016/j.foodchem.2020.127819. 32810812

[ref101] Mallhi Z. I.; Rizwan M.; Mansha A.; Ali Q.; Asim S.; Ali S.; Hussain A.; Alrokayan S. H.; Khan H. A.; Alam P.; Ahmad P. Citric Acid Enhances Plant Growth, Photosynthesis, and Phytoextraction of Lead by Alleviating the Oxidative Stress in Castor Beans. Plants 2019, 8 (11), 525 10.3390/plants8110525. 31752443 PMC6918418

[ref102] Tusei C. The Effects of Citric Acid on pH and Nutrient Uptake in Wheatgrass (Triticum aestivum). https://digitalcommons.humboldt.edu/ideafest/vol3/iss1/7 (accessed July 12, 2024).

[ref103] Donovan C.; Sun M.; Cogswell D.; Margo C. E.; Avila M. Y.; Espana E. M. Genipin increases extracellular matrix synthesis preventing corneal perforation. Ocul. Surf. 2023, 28, 115–123. 10.1016/j.jtos.2023.02.003. 36871831 PMC10440284

[ref104] Heimbuck A. M.; Priddy-Arrington T. R.; Padgett M. L.; Llamas C. B.; Barnett H. H.; Bunnell B. A.; Caldorera-Moore M. E. Development of Responsive Chitosan-Genipin Hydrogels for the Treatment of Wounds. ACS Appl. Bio Mater. 2019, 2 (7), 2879–2888. 10.1021/acsabm.9b00266. 35030822

[ref105] Huang Y. Y.; Yao Q. B.; Jia X. Z.; Chen B. R.; Abdul R.; Wang L. H.; Zeng X. A.; Liu D. M. Characterization and application in yogurt of genipin-crosslinked chitosan microcapsules encapsulating with Lactiplantibacillus plantarum DMDL 9010. Int. J. Biol. Macromol. 2023, 248, 125871 10.1016/j.ijbiomac.2023.125871. 37473896

[ref106] Raja I. S.; Fathima N. N. Gelatin-Cerium Oxide Nanocomposite for Enhanced Excisional Wound Healing. ACS Appl. Bio Mater. 2018, 1 (2), 487–495. 10.1021/acsabm.8b00208. 35016389

[ref107] Ahmed R.; ul ain Hira N.; Wang M.; Iqbal S.; Yi J.; Hemar Y. Genipin, a natural blue colorant precursor: Source, extraction, properties, and applications. Food Chem. 2024, 434, 137498 10.1016/j.foodchem.2023.137498. 37741231

[ref108] Ghaly A.; Ramakrishnan V. Nitrification of urea and assimilation of nitrate in saturated soils under aerobic conditions. Am. J. Agric. Biol. Sci. 2013, 8, 330–342. 10.3844/ajabssp.2013.330.342.

[ref109] Ustin S. L.; Gitelson A.; Jacquemoud S.; Schaepman M. E.; Asner G.; Gamon J.; Zarco-Tejada P. Retrieval of foliar information about plant pigment systems from high resolution spectroscopy. Remote Sens. Environ. 2009, 113, S67–S77. 10.1016/j.rse.2008.10.019.

[ref110] Tahjib-Ul-Arif M.; Zahan M. I.; Karim M. M.; Imran S.; Hunter C. T.; Islam M. S.; Mia M. A.; Hannan M. A.; Rhaman M. S.; Hossain M. A.; Brestic M.; Skalicky M.; Murata Y. Citric Acid-Mediated Abiotic Stress Tolerance in Plants. Int. J. Mol. Sci. 2021, 22 (13), 7235 10.3390/ijms22137235. 34281289 PMC8268203

[ref111] Afshan S.; Ali S.; Bharwana S. A.; Rizwan M.; Farid M.; Abbas F.; Ibrahim M.; Mehmood M. A.; Abbasi G. H. Citric acid enhances the phytoextraction of chromium, plant growth, and photosynthesis by alleviating the oxidative damages in Brassica napus L. Environ. Sci. Pollut. Res. Int. 2015, 22 (15), 11679–11689. 10.1007/s11356-015-4396-8. 25850739

[ref112] Farid M.; Ali S.; Rizwan M.; Ali Q.; Abbas F.; Bukhari S. A. H.; Saeed R.; Wu L. Citric acid assisted phytoextraction of chromium by sunflower; morpho-physiological and biochemical alterations in plants. Ecotoxicol. Environ. Saf. 2017, 145, 90–102. 10.1016/j.ecoenv.2017.07.016. 28710950

[ref113] Maoka T. Carotenoids as natural functional pigments. J. Nat. Med. 2020, 74 (1), 1–16. 10.1007/s11418-019-01364-x. 31588965 PMC6949322

[ref114] Martins T.; Barros A. N.; Rosa E.; Antunes L. Enhancing Health Benefits through Chlorophylls and Chlorophyll-Rich Agro-Food: A Comprehensive Review. Molecules 2023, 28 (14), 5344 10.3390/molecules28145344. 37513218 PMC10384064

[ref115] Bolhàr-Nordenkampf H.; Grünweis E. Determination of the total chlorophyll distribution pattern in living leaves. Photosynth. Res. 1987, 12 (1), 13–23. 10.1007/BF00019147. 24435577

[ref116] Shibghatallah M.; Khotimah S.; Suhandono S.; Viridi S.; Kesuma T. Measuring Leaf Chlorophyll Concentration from Its Color: A Way in Monitoring Environment Change to Plantations. AIP Conf. Proc. 2013, 1554, 210–213. 10.1063/1.4820322.

[ref117] Yadava U. L. A Rapid and Nondestructive Method to Determine Chlorophyll in Intact Leaves. HortScience 1986, 21 (6), 1449–1450. 10.21273/HORTSCI.21.6.1449.

[ref118] Björn L. O.; Papageorgiou G. C.; Blankenship R. E.; Govindjee Govindjee, A viewpoint: why chlorophyll a?. Photosynth. Res. 2009, 99, 85–98. 10.1007/s11120-008-9395-x. 19125349

[ref119] Espinoza C. M.; Reyna M. A. ´. V. Mecanismos de respuesta al estrés abiótico: hacia una perspectiva de las especies forestales. Revista Mexicana de Ciencias Forestales. 2019, 10 (56), 33–64. 10.29298/rmcf.v10i56.567.

[ref120] Badhani B.; Sharma N.; Kakkar R. Gallic Acid: A Versatile Antioxidant with Promising Therapeutic and Industrial Applications. RSC Advances 2015, 5, 27540–27557. 10.1039/C5RA01911G.

[ref121] Nunes C.; Maricato E. ´.; Cunha A. ^.; Nunes A.; Silva J. A. L. d.; Coimbra M. A. Chitosan-caffeic acid-genipin films presenting enhanced antioxidant activity and stability in acidic media. Carbohydr. Polym. 2013, 91 (1), 236 10.1016/j.carbpol.2012.08.033. 23044128

[ref122] Cho Y. S. Genipin, an Inhibitor of UCP2 as a Promising New Anticancer Agent: A Review of the Literature. Int. J. Mol. Sci. 2022, 23 (10), 5637–5713. 10.3390/ijms23105637. 35628447 PMC9147402

[ref123] Khosravi F.; H M.; Azizi; Rabani M.; Nadoshan R. M. Assessment of the biotechnological activity of wheat hydrolysates prepared with the Biarum bovei extract. J. Food Meas. Char. 2022, 16 (4), 2738–2748. 10.1007/s11694-022-01379-1.

[ref124] Ye X.; Capezza A. J.; Gowda V.; Olsson R.; Lendel C.; Hedenqvist M. High-Temperature and Chemically Resistant Foams from Sustainable Nanostructured Protein. Adv. Sustainable Syst. 2021, 5, 1–9. 10.1002/adsu.202100063.

[ref125] Lai S.; Cheng C.; Yuan B.; Liao Y.; Su X.; Bai S. Mechanochemical reclaiming and thermoplastic re-processing of waste Acrylonitrile-butadiene rubber (NBR)/poly (Vinyl Chloride) (PVC) insulation materials. J. Waste Manag. 2023, 158, 153–163. 10.1016/j.wasman.2023.01.019. 36709681

[ref126] Jiménez-Rosado M.; Zarate-Ramírez L. S.; Romero A.; Bengoechea C.; Partal P.; Guerrero A. Bioplastics based on Wheat Gluten processed by Extrusion. J. Clean. Prod. 2019, 239 (117994), 117994 10.1016/j.jclepro.2019.117994.

[ref127] Afshar S. V.; Boldrin A.; Astrup T. F.; Daugaard A. E.; Hartmann N. B. Degradation of biodegradable plastics in waste management systems and the open environment: A Critical review. J. Clean. Prod. 2024, 434, 140000 10.1016/j.jclepro.2023.140000.

