# Supplementary material for: Biodegradation, Bioassimilation and Recycling Properties of Wheat Gluten Foams
Source: ACS Agric Sci Technol. 2025 Apr 4;5(5):805–21. doi: 10.1021/acsagscitech.4c00798 (PMC12093299; doi:10.1021/acsagscitech.4c00798)
Supplement: Supplementary file 1 — as4c00798_si_001.pdf [file as4c00798_si_001.pdf]

Supplementary information

Biodegradation, Bioassimilation and Recycling Properties of Wheat Gluten Foams

Mercedes A. Bettelli[1]*, Leonardo A. Perdigón[2], Luyao Zhao[3], Pamela F.M. Pereira[4], Amparo Jiménez-Quero[4], Antonio J. Capezza[1], Thomas Prade[5], Eva Johansson[6], Richard T. Olsson[1], Mikael S. Hedenqvist[1]*, Marcos A. Sabino[2]

1 Department of Fibre and Polymer Technology, Polymeric Materials Division, School of Engineering Sciences in Chemistry, Biotechnology and Health. KTH Royal Institute of Technology, 100 44 Stockholm, Sweden.

2 Department. of Chemistry, B5IDA research group, Simon Bolivar University, Caracas 89000, Venezuela.

3 Department of Industrial Biotechnology, School of Engineering Sciences in Chemistry, Biotechnology and Health. KTH Royal Institute of Technology, 100 44 Stockholm, Sweden.

4 Department of LIFE Sciences, Industrial Biotechnology Division, Chalmers University of Technology, 412 96 Gothenburg, Sweden.

5 Department of Biosystems and Technology, Swedish University of Agricultural Sciences, Box 190, 234 22 Lomma, Sweden.

6 Department of Plant Breeding, The Swedish University of Agricultural Sciences, Box 190, 234 22 Lomma, Sweden.

Number of pages: 26
Number of figures: 21
Number of tables: 4

Table S1. Components used in a foamed nitrile butadiene rubber (NBR).

**Component**	**For 1 kg of foamed NBR (g)**
NBR (Paracril 34PE40N)	370
Polyvinyl chloride resin powder	200
Bis(2-ethylhexyl) phthalate (softener)	300
Calcium carbonate (pigment)	80
Ca-Zn$(CoCR)_2$ (heat stabilizer)	25
Azodicarbonamide (whipping agent)	25
**Total**	**1000**

Patent: CN103351489A. Preparation method of nitrile butadiene rubber yoga mat. (2013). https://patents.google.com/patent/CN103351489A/en, 29/05/2024

Table S2: Global warming potential (GWP) for the compounds used in the production of the foams as well as carbon content, the proportion of renewable carbon, and corresponding GWP calculated from the amount of fossil carbon, assuming complete mineralization

Compound	GWP, material (kg CO_2e/kg)	Reference	Carbon content (%)	Renewable carbon (%)	GWP, combustion (%)
NBR (copolymer-acrylonitrile-butadiene)	2970	Ecoinvent 3.9.1 - Market for synthetic rubber	78.5	0	2877
Polyvinyl chloride resin	3050	Ecoinvent 3.10 - Market for polyvinyl chloride	38.4	0	1409
Wheat gluten (WG)	1551	[1]	n/d	100	0
Glycerol	2690	Ecoinvent 3.9.1 - Market for glycerine	39.1	100	0
Ammonium bicarbonate	1270	Ecoinvent 3.9.1 - Market for ammonium bicarbonate	15.2	0	557
Calcium carbonate (colorant)	812	Ecoinvent 3.9.1 - Market for calcium carbonate	40.0	0	1468
Gallic acid (cross-linker)	1515	[1]	49.4	100	0
Citric acid (cross-linker)	5940	Ecoinvent 3.9.1 - Market for citric acid	37.5	100	0
Genipin[a,b]	n/d		58.4	100	0
Bis(2-ethylhexyl) phthalate (softener)	3779	[2]	67.9	0,0	2489
Azodicarbomide (foaming agent)	13200	Ecoinvent 3.9.1 - Market for azodicarbomide	20.7	0,0	13200
Ca-Zn(CoCR)$_2$, heat stabiliser[c]	723	Ecoinvent 3.10 - Market data	86.8	100	0

[a] No data available.

[b] The estimations of the GWP did not include the impact of the use of genipin in alternative foam material formulations WG/G/ABC/1GNP and WG/G/ABC/5GNP due to lack of data. Genipin is a non-cytotoxic crosslinker extracted from the genipap fruit (*Genipa american* L.) or Gardenia (*Gardenia jasminoides*).[3] While extracts from Gardenia require an additional enzymatic hydrolysis of the component geniposide for genipin [4], oil extracts from the genipap fruit contain genipin in high concentrations [5]. Both fruits and wood are the focus of the commercialization of the cultivation of the genipap tree.[6] To our knowledge, the impact of genipin production and the resulting climate effect has not been studied so far. While the impact is unknown, only relatively small amounts of genipin (1 and 5%) are used in the two materials tested that contain genipin. The currently high prices per genipin of about 3000 €/kg are, however, restricting large-scale use of it. Commercialization of genipin may require the use of less refined genipin, e.g. through direct use of genipap oil.[5]

[c] No data available. Assumed as synthesis from stearic acid, sodium hydroxide, and metal salts (calcium chloride, zinc sulfate) as suggested in ref.[7] Only material use was considered, and emissions from the processing were disregarded.

References in Table S2

1. Shinde, P. N.; Mandavgane, S. A.; Karadbhajane, V., Process development and life cycle assessment of pomegranate biorefinery. *Environ Sci Pollut Res.* **2020,** *27* (20), 25785-25793.
2. Li, Y. 2013 Life Cycle Assessment to Di-2-Ethylhexyl Phthalate (DEHP), applications and potential alternatives. University of Pittsburgh, 2013.
3. Ahmed, R.; ul ain Hira, N.; Wang, M.; Iqbal, S.; Yi, J.; Hemar, Y., Genipin, a natural blue colorant precursor: Source, extraction, properties, and applications. *Food Chem.* **2024,** *434*, 137498.
4. Ramos-de-la-Peña, A. M.; Renard, C. M. G. C.; Wicker, L.; Montañez, J. C.; García-Cerda, L. A.; Contreras-Esquivel, J. C., Environmental friendly cold-mechanical/sonic enzymatic assisted extraction of genipin from genipap (Genipa americana). *Ultrason Sonochem.* **2014,** *21* (1), 43-49.
5. Hurtado Colmenares, L. B.; Nejati, M.; Fang, Y.; Guo, B.; Jiménez-Quero, A.; Capezza, A. J.; Sabino, M. A., New sources of genipin-rich substances for crosslinking future manufactured bio-based materials. *RSC Sustainability* **2024,** *2* (1), 125-138.
6. Faria, T. A. C.; Curi, T. M. R. d. C.; Santo, T. L. d. E.; Vieira, G. H. d. C.; Costa, E.; Larson, L. C. R. d. S.; Binotti, F. F. d. S., Substrates and cultivation environments in the production of seedlings of Genipa americana L. (Rubiaceae) seedlings. *Rese Soc Dev.* **2020,** *9* (10), e5759107920.
7. Putrawan, I. D. G. A.; Indarto, A.; Octavia, Y., Thermal stabilization of polyvinyl chloride by calcium and zinc carboxylates derived from byproduct of palm oil refining. *Heliyon.* **2022,** *8* (8), e10079.

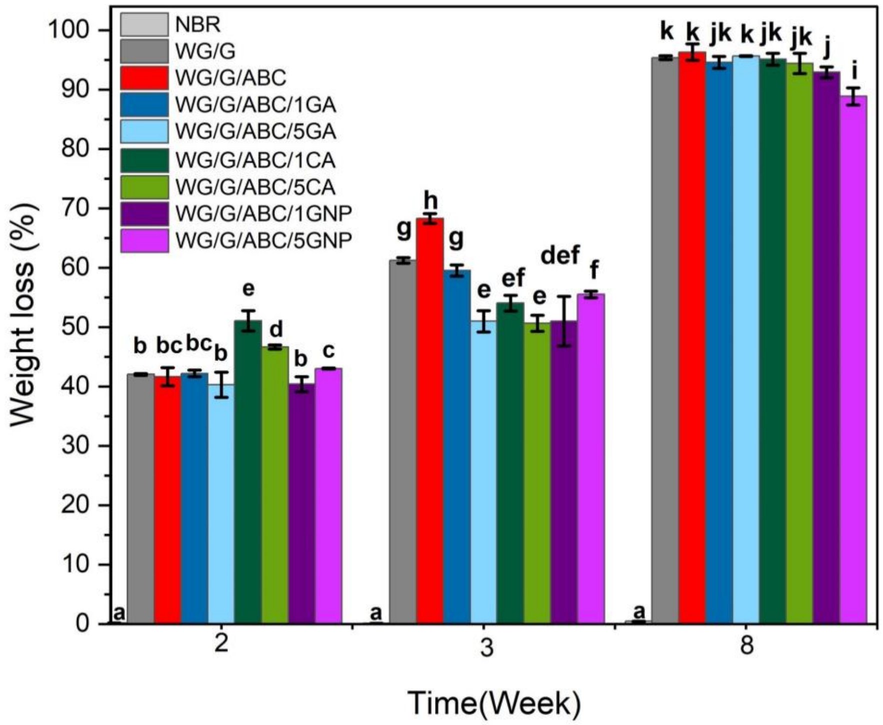

Figure S1. Biodegradation in soil: (a) weight loss after 2, 3, and 8 weeks. Note: Different letters mean the values are significantly different (P <0.05).

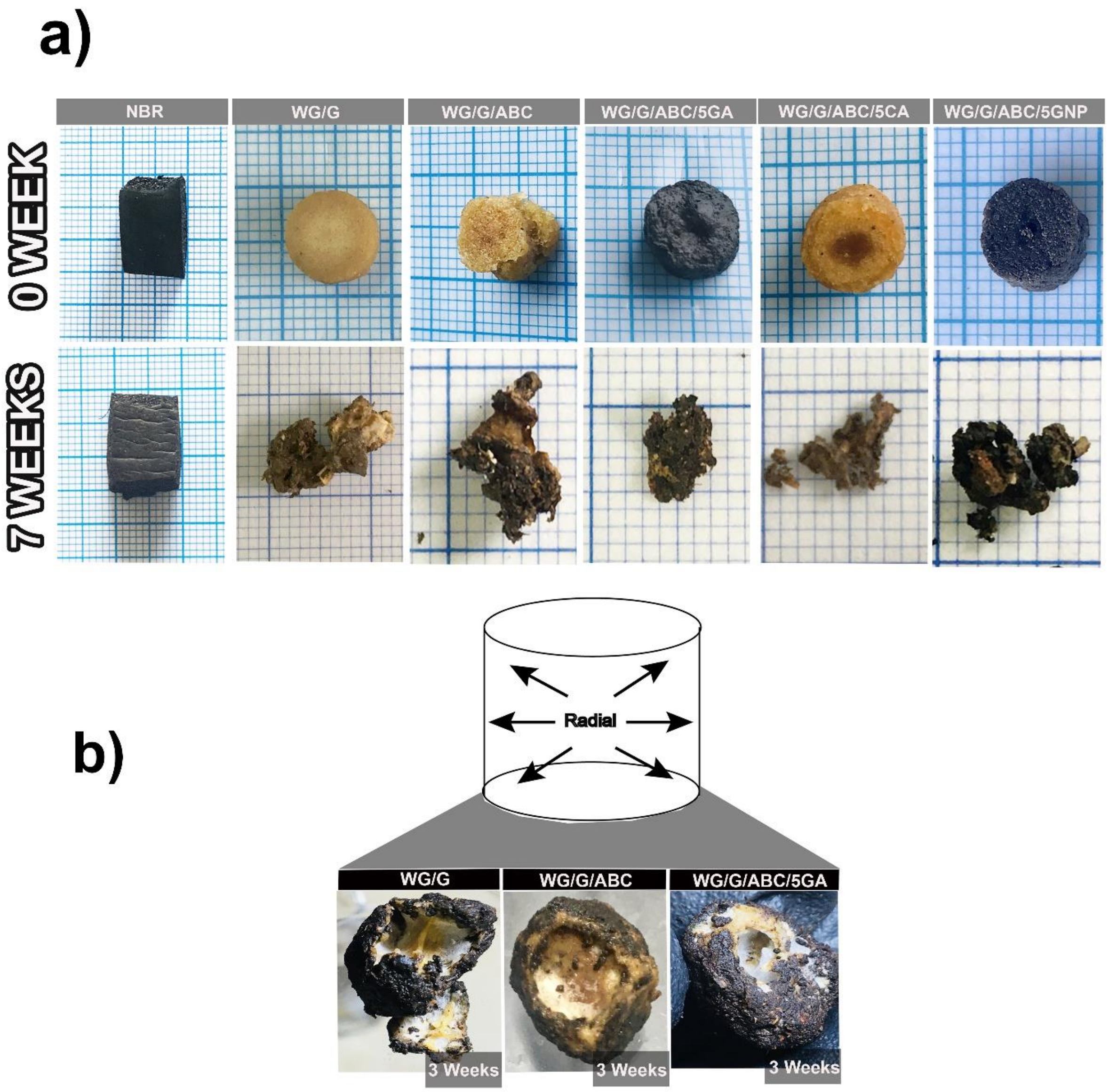

Figure S2. Biodegradation in soil: (a) cross-section appearance before and after degradation, and (b) degradation along foam-rod radius.

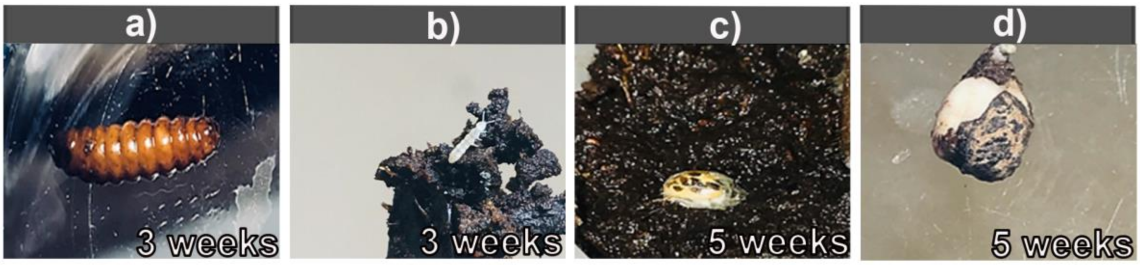

Figure S3. Species found during soil degradation. (a) larvae, (b) insect, (c) insect feces. (d) The endosperm of germinating seed.

Video S1. Microbial activity during soil degradation.

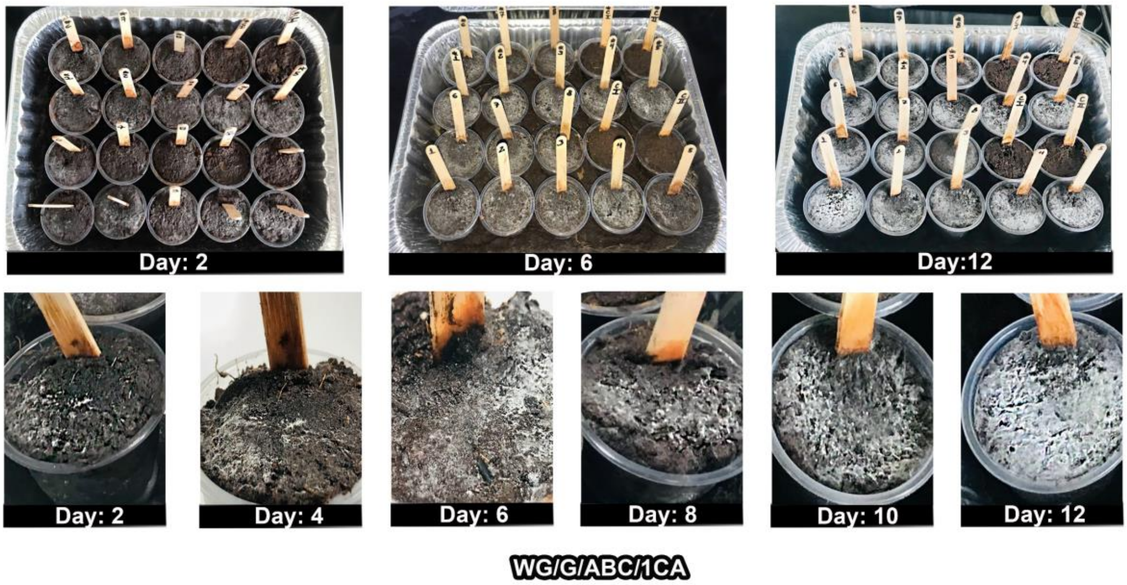

Figure S4. Development of mold colonies as a function of time for 1wt.% CA during soil degradation.

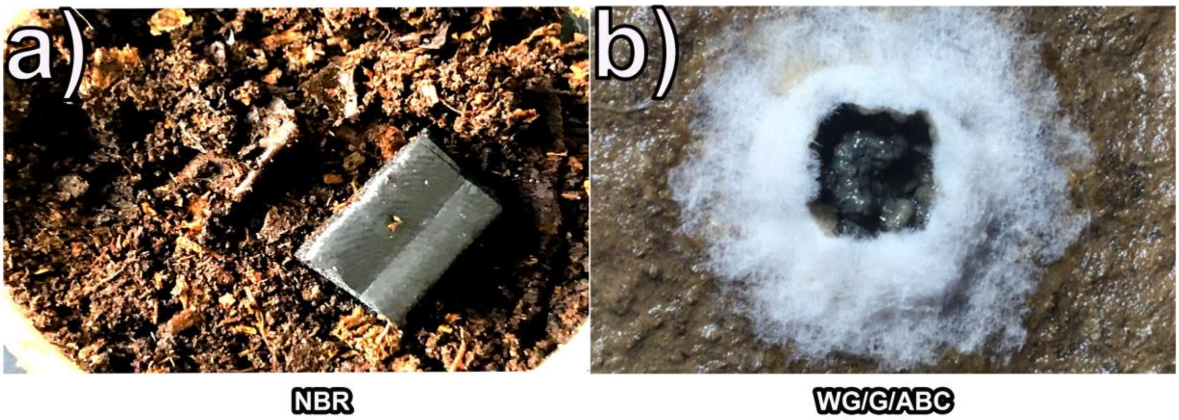

Figure S5. (a) NBR and (b) WG/G/ABC foam placed on top of the soil for 2 weeks. No microbial activity was observed in the NBR case, but fungal growth was evident in the WG case.

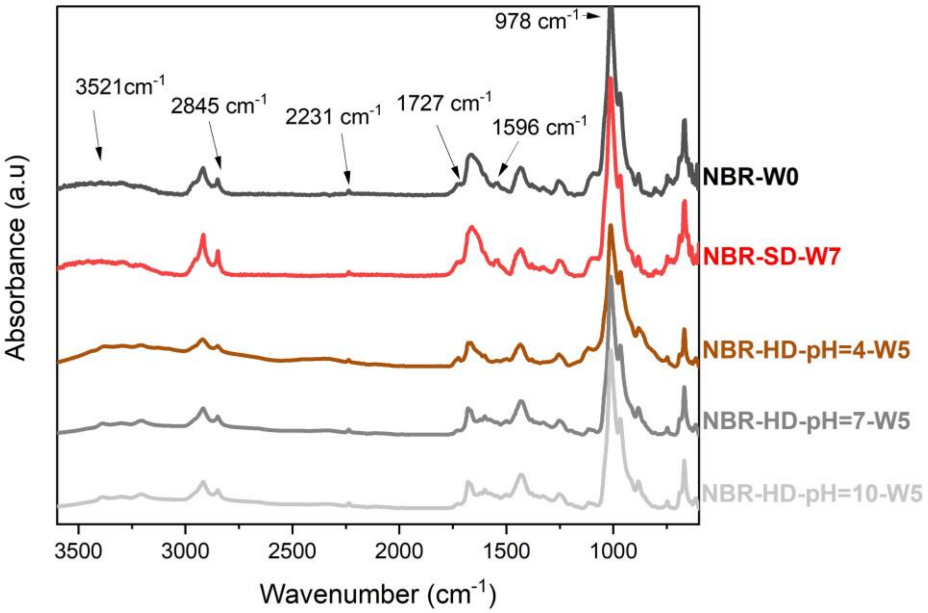

Figure S6. Full FTIR spectra of the NBR foam in different environments.

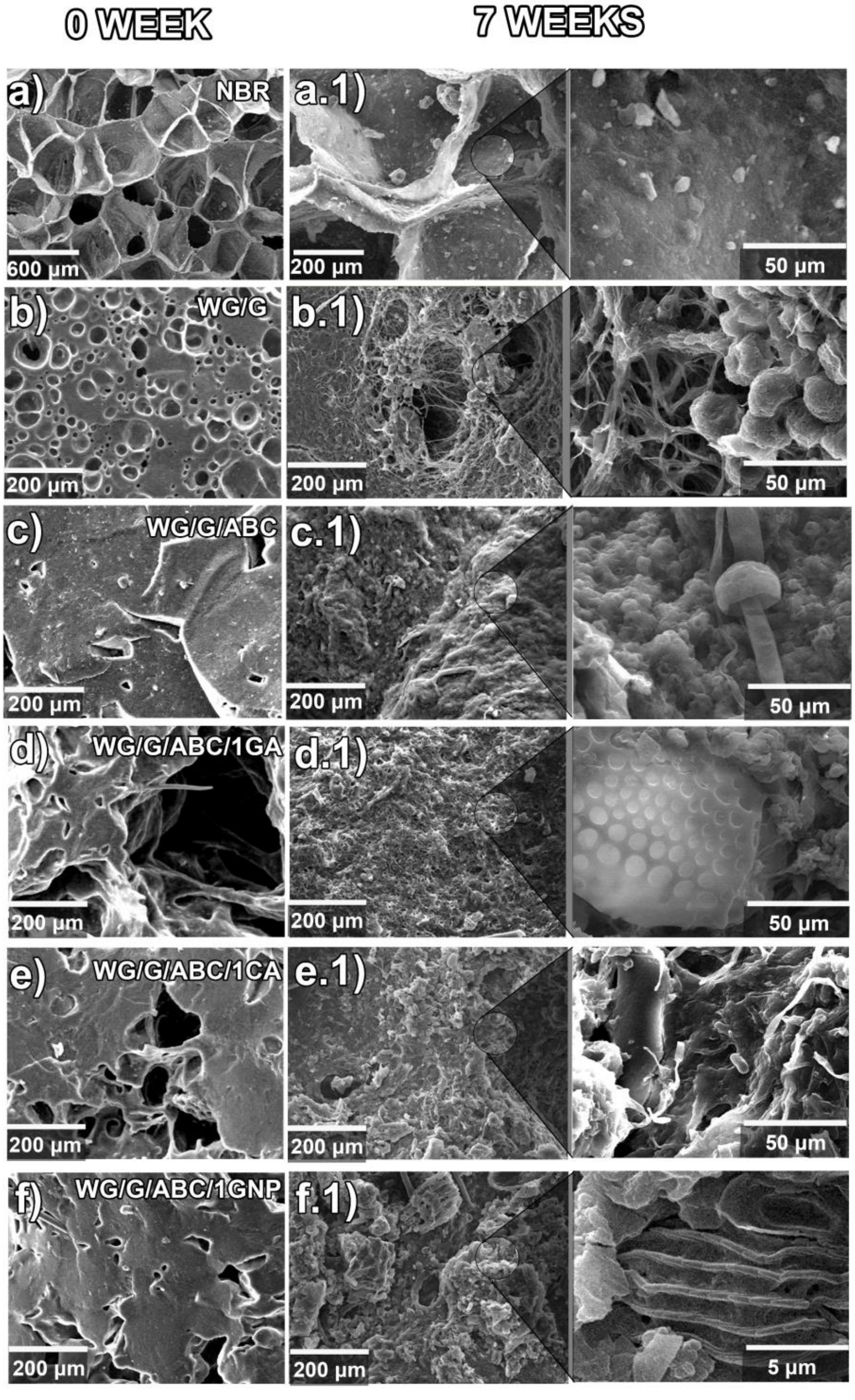

Figure S7. SEM cross-sections of extruded samples after 7 weeks in soil.

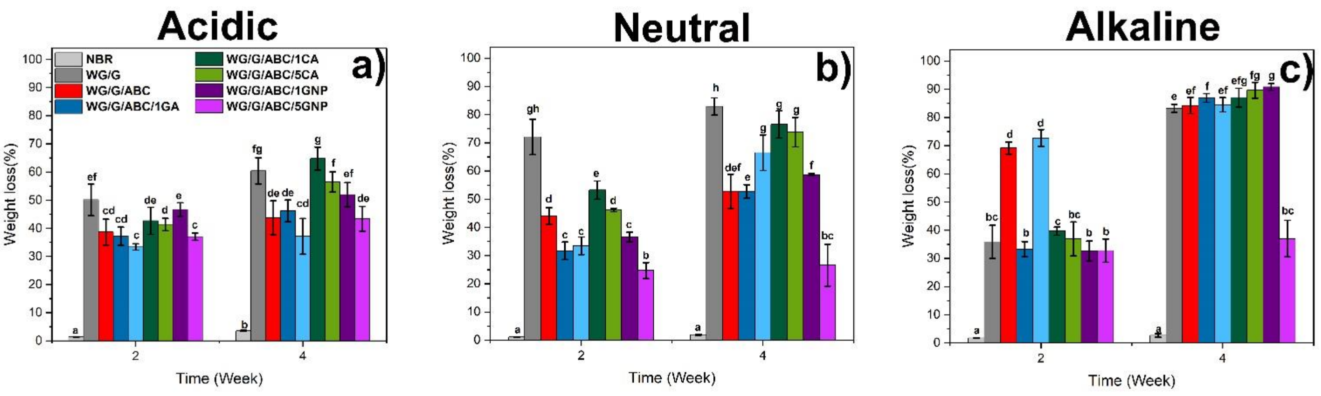

Figure S8. Weight loss after 2 and 4 weeks in acidic, neutral, and alkaline conditions. Note: Different letters mean the values are significantly different (P <0.05).

Table S3: Weight loss of WG foams under different degradation conditions.

Samples	Soil degradation (%)		Hydrolytic degradation (%)					
	pH=NC[1]		pH=Acidic		pH=Neutral		pH=Alkaline	
	Week							
	1	5	1	5	1	5	1	5
**NBR**	0.17±0.001^{a}	0.15±0.02^{a}	0.9±0.2^{a}	3.8±0.5^{b}	0.5±0.2^{a}	2.9±0.4^{b}	0.9±0.1^{a}	3.5±0.4^{b}
**WG/G**	33.2±0.8 ^{d}	73.3±0.5^{g}	34.9±3.1^{f}	62.4±6.8^{ijk}	40.1±2.7^{e}	88.5±4.2^{i}	28.4±3.5^{c}	88.6±2.4^{i}
**WG/G/ABC**	31.5±0.2 ^{d}	83.4±2.6^{i}	18.4±1.6^{d}	45.6±5.8^{hi}	28.9±3.4^{cd}	67.0±2.7^{h}	30.8±2.5^{cd}	90.9±1.4^{i}
**WG/G/ABC/1GA**	25.5±0.2 ^{b}	79.0±0.4^{h}	25.2±2.6^{e}	52.0±4.7^{i}	28.2±2.6^{cd}	74.3±6.5^{hi}	26.9±2.9^{c}	90.5±2.1^{i}
**WG/G/ABC/5GA**	30.69±0.4 ^{c}	65.6±2.5^{f}	29.2±2.0^{e}	43.0±6.8^{fhi}	27.7±2.7^{cd}	75.3±6.4^{hi}	43.7±3.4^{e}	90.6±2.0^{i}
**WG/G/ABC/1CA**	33.0±2.7 ^{cd}	81.4±2.6^{hi}	12.7±3.3^{c}	69.9±1.6^{k}	31.7±2.0^{d}	84.2±5.4^{i}	31.3±3.8^{cd}	91.7±2.3^{ij}
**WG/G/ABC/5CA**	37.2±0.5 ^{e}	57.4±6.1^{f}	40.0±3.3^{f}	63.0±1.8^{j}	33.0±2.5^{d}	95.0±7.3^{i}	36.1±2.2^{d}	98.6±2.6^{i}
**WG/G/ABC/1GNP**	35.2±2.3 ^{de}	70.5±7.4^{fg}	44.7±1.6^{h}	53.3±5.67^{i}	30.0±2.8^{d}	60.6±1.2^{g}	29.5±2.5^{c}	95.2±1.7^{j}
**WG/G/ABC/5GNP**	32.1±0.6 ^{d}	64.3±1.0^{f}	35.6±2.8^{f}	44.6±5.7^{fhi}	23.2±2.2^{c}	31.8±8.3^{cde}	29.8±2.9^{c}	60.4±2.4^{f}

Note: [1] A non-controlled environment was used during the testing (pH, temperature, uv-light, etc). Note: Different letters mean the values are significantly different (P <0.05).

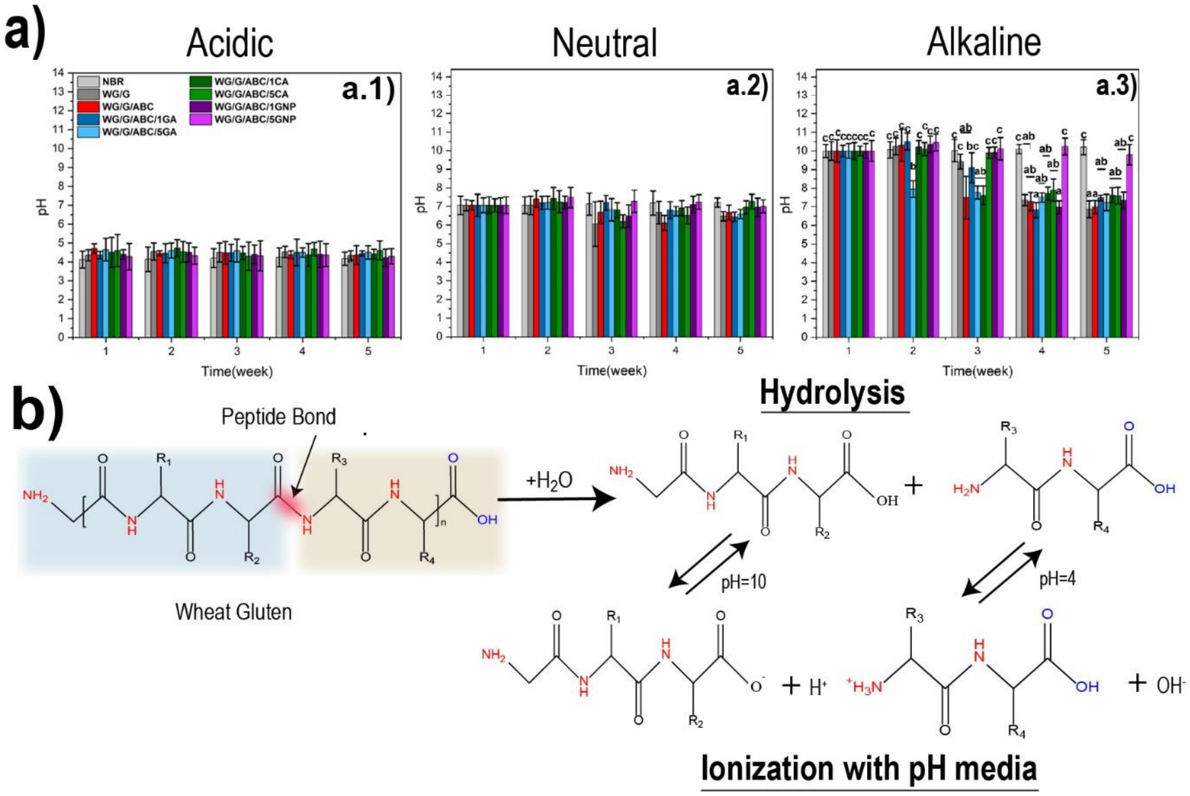

Figure S9. (a) pH of the buffer solutions in the different sample systems. (b) Hydrolysis of peptides showing ionization at pH 4 and pH 10. The values in a.1 and a.2 are not significantly different (P <0.05). In a.3 different letters mean the values are significantly different (P <0.05).

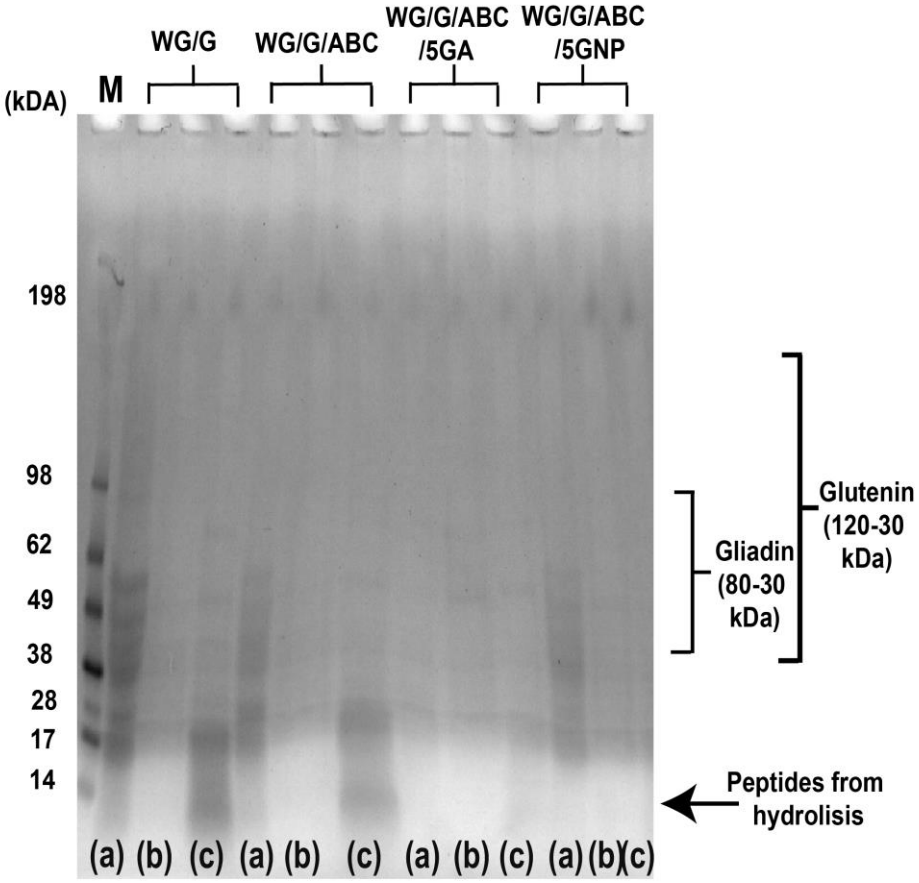

Figure S10. Electropherogram of WG/G, WG/G/ABC, WG/G/ABC/5GA, and WG/G/ABC/5GNP after 4 weeks in (a) acidic, (b) neutral and (c) alkaline conditions, showing the banding pattern and molecular weight marker.

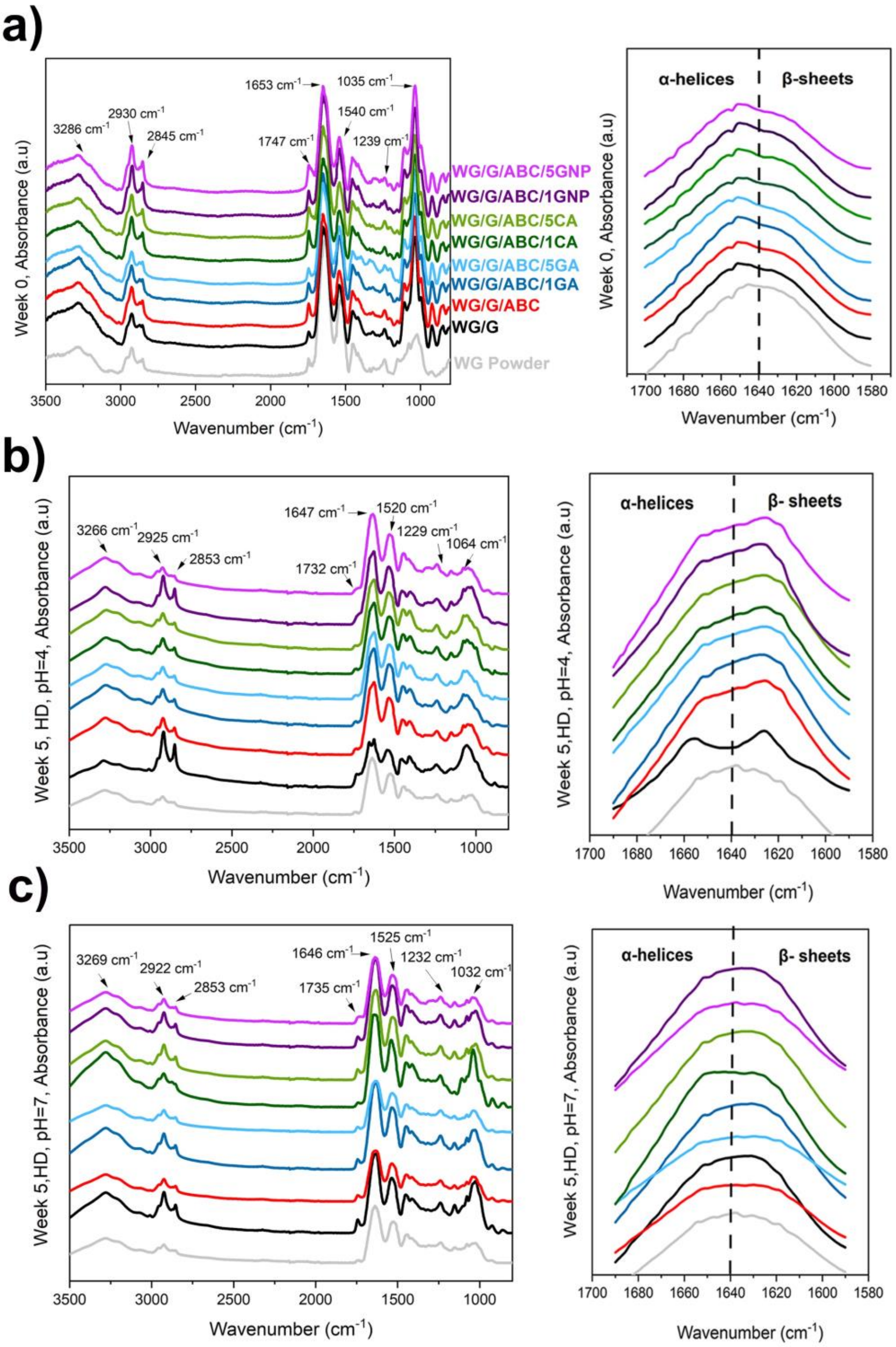

Figure S11. Full FTIR spectra and their respective amide I region (1700-1580 cm^{-1}) of the samples under hydrolytic degradation: (a) before degradation, (b) hydrolytic degradation (HD) at pH 4 and (c) pH 7 after 5 weeks.

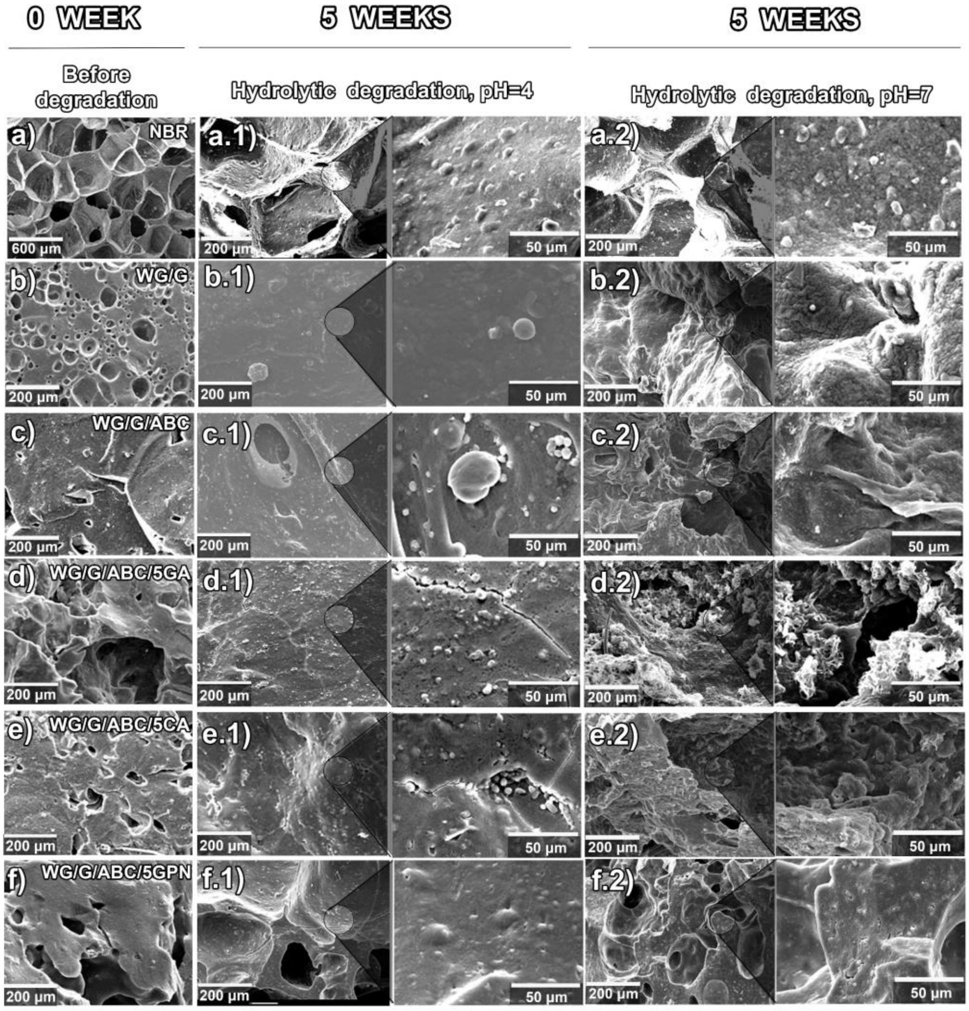

Figure S12. Cross-sections of extruded samples were revealed by SEM before and after hydrolytic degradation for 5 weeks.

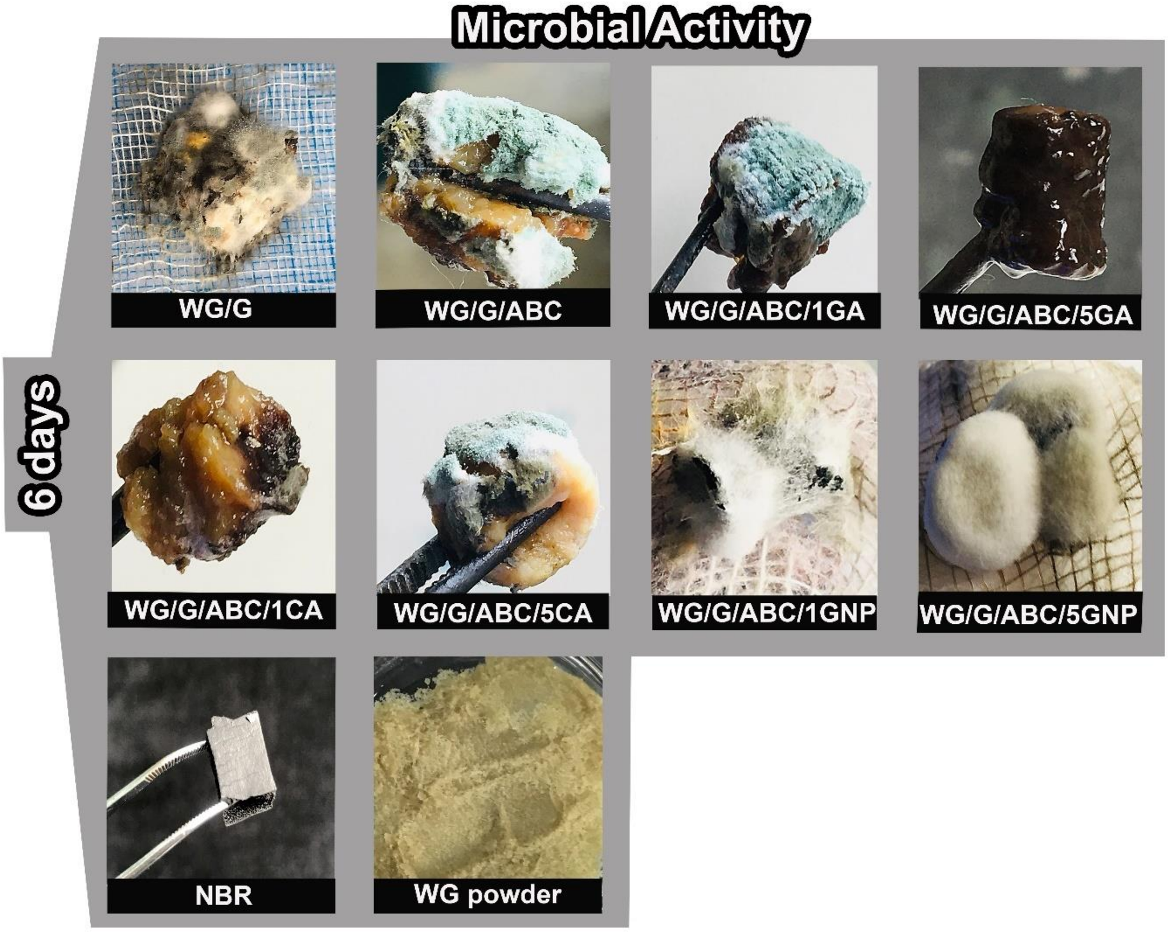

Figure S13: Microbial activity in samples after 6 days during high humidity environment.

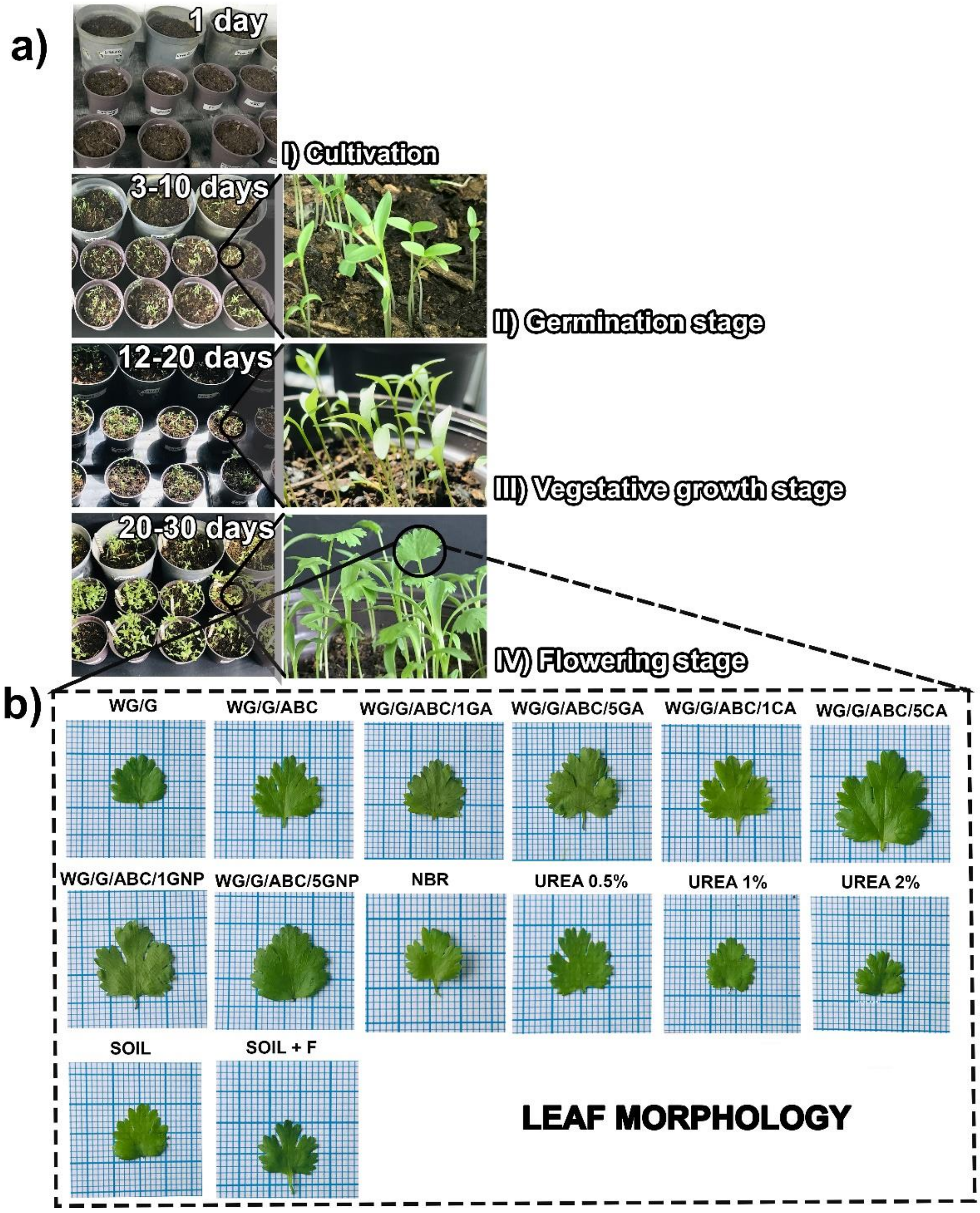

Figure S14. a) Different stages of coriander growth and b) typical leaf morphologies.

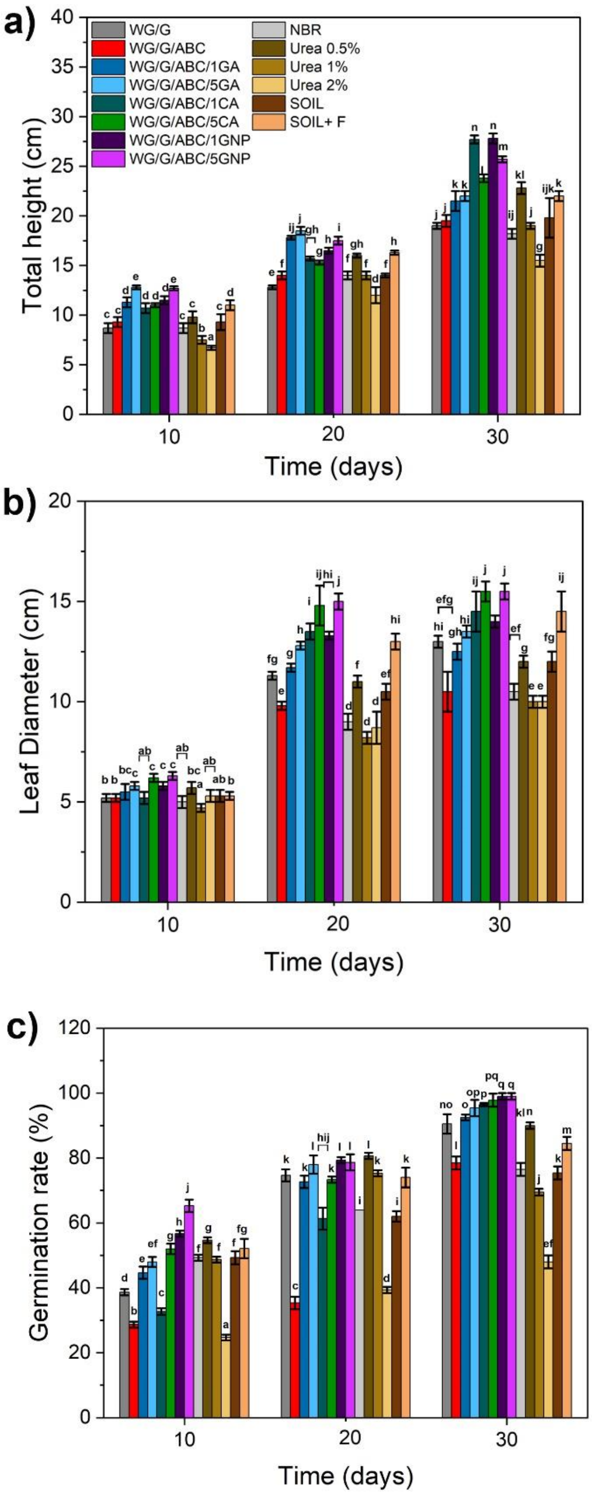

Figure S15. Indicators of vegetal growth: a) Total height, b) leave diameters, and c) germination rate. Different letters mean the values are significantly different (P <0.05).

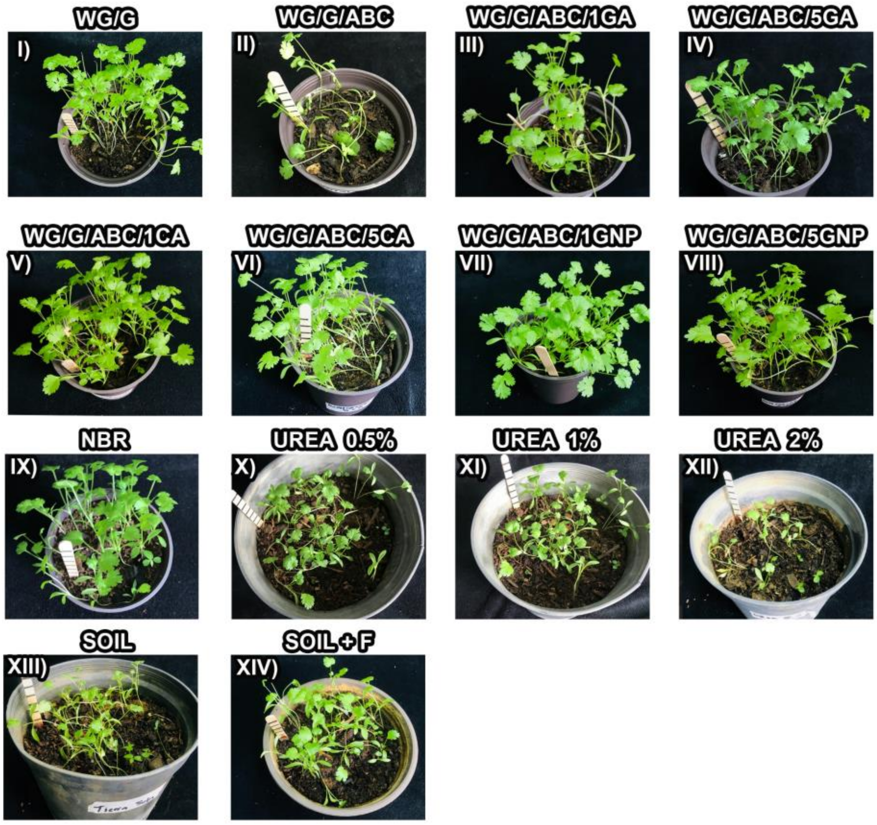

Figure S16. Vegetative growth after 20 days of cultivation.

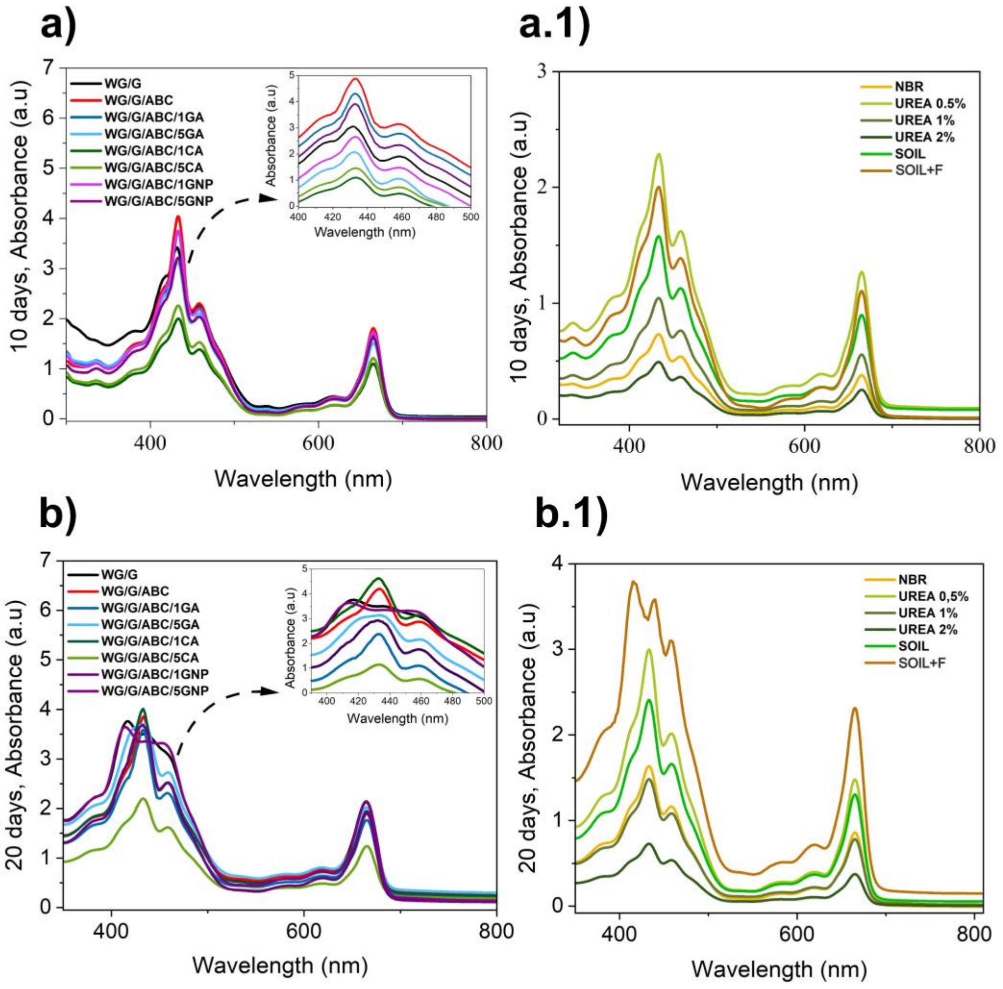

Figure S17. UV spectra (at 450-480 and 640-670 nm) of coriander plant after (a, a.1) 10 and (b, b.1) 20 days of germination.

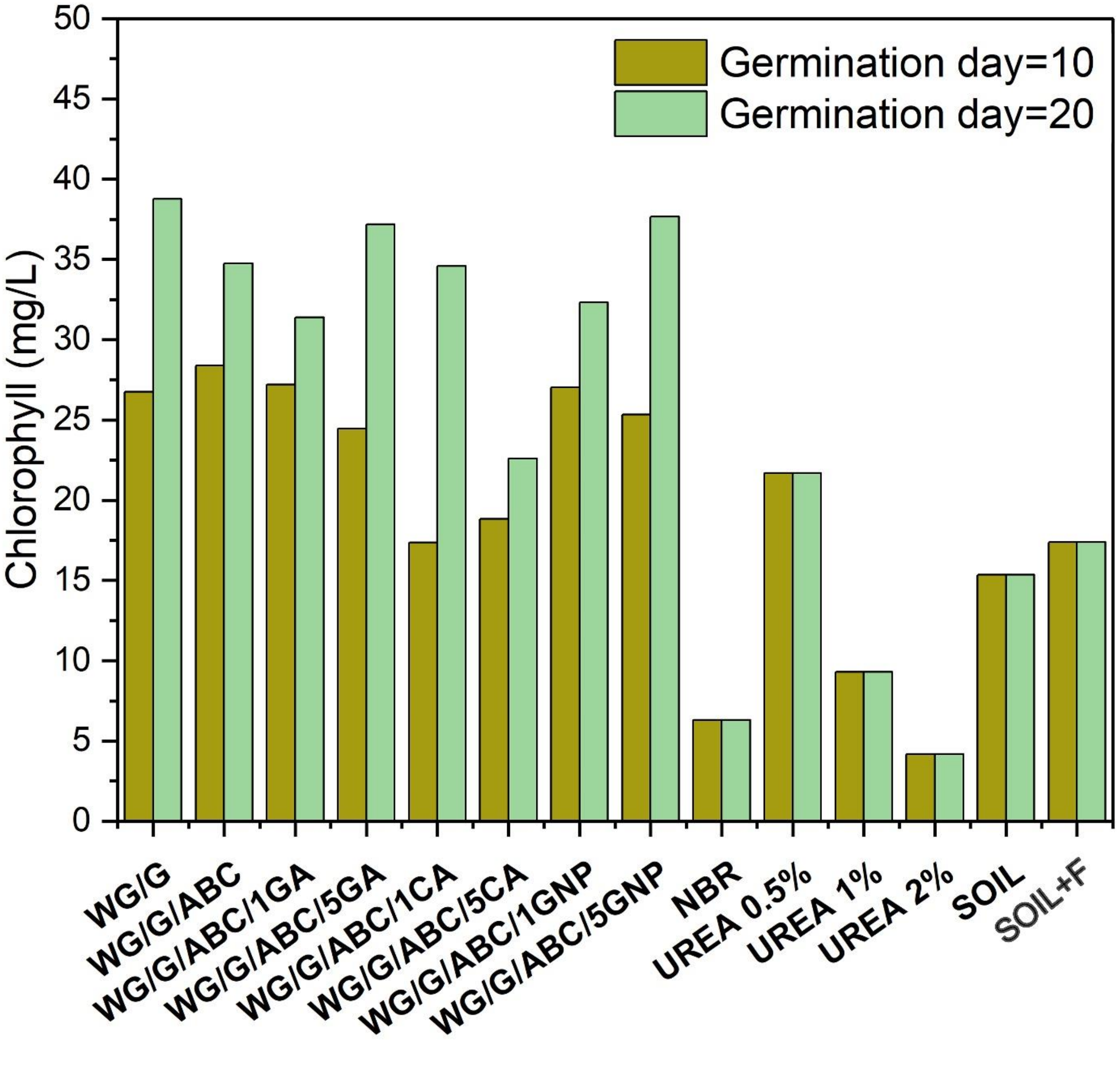

Figure S18. Content of chlorophyll pigment in the coriander leaves after 10 and 20 days of germination.

Table S4. Radical scavenging activity of WG and additives used, expressed as EC50.

**Additives**	**EC50 (mg/mg of DPPH)**
ABC	N.D.
SBC	N.D.
CA	N.D.
GA	0.0773
WG	61.67
GNP	293.37

Note: N.D: Not detected.

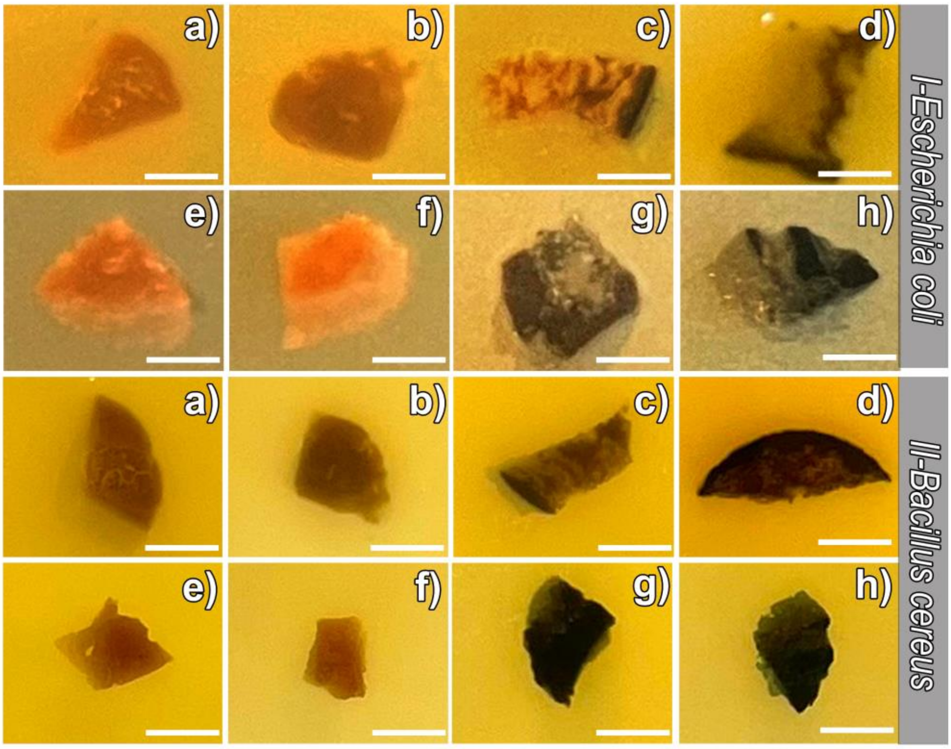

Figure S19. Antibacterial activity of a) WG/G, b) WG/G/ABC, c) WG/G/ABC/1GA, d) WG/G/ABC/5GA, e) WG/G/ABC/1CA, f) WG/G/ABC/5CA, g) WG/G/ABC/1GNP, and h) WG/G/ABC/5GNP materials against (I) *E. coli* and (II) *B. cereus*, respectively. The scale bar is 0.5 cm**.**

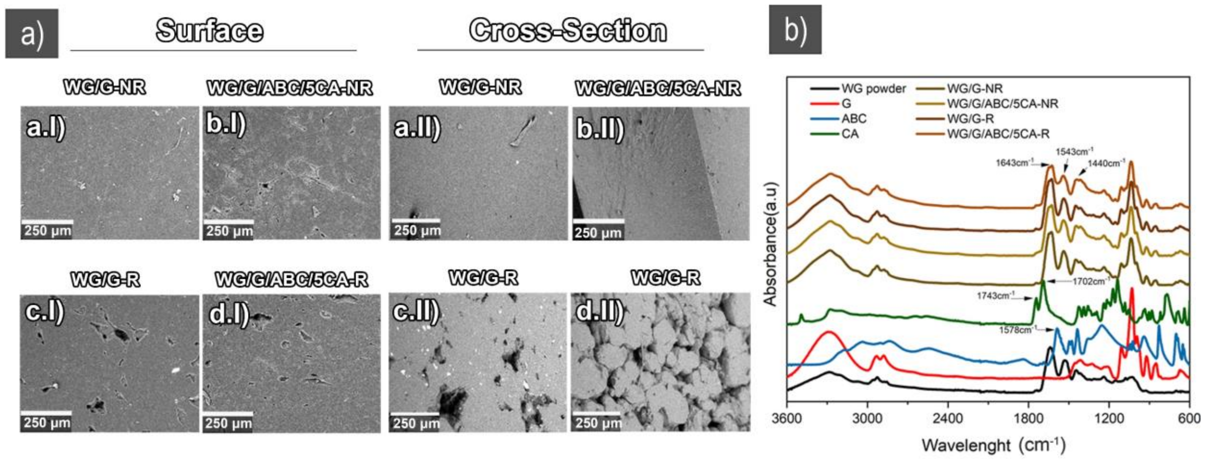

Figure S20. (a) Microstructure of non-recycled (NR) and recycled (R) compression-moulded sheets of WG/G and WG/G/ABC/5CA, revealed by SEM. (b) Full FTIR spectra of non-recycled (NR) and recycled (R) samples and the pure additives.

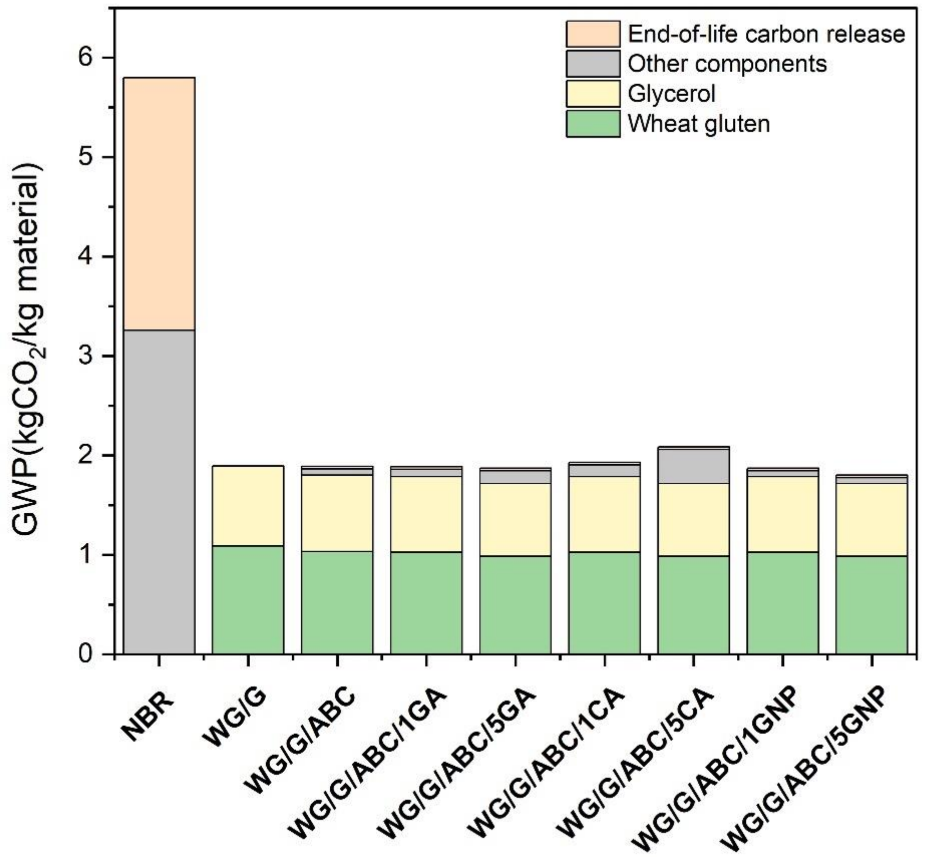

Figure S21. Global warming potential and end-of-life fossil carbon release.
